# Field effect transistor based wearable biosensors for healthcare monitoring

**DOI:** 10.1186/s12951-023-02153-1

**Published:** 2023-11-07

**Authors:** Thi Thanh-Ha Nguyen, Cong Minh Nguyen, Minh Anh Huynh, Hoang Huy Vu, Tuan-Khoa Nguyen, Nam-Trung Nguyen

**Affiliations:** 1 Queensland Micro- and Nanotechnology Centre, Griffith University, Nathan, QLD 4111 Australia; 2 School of Engineering and Built Environment, Griffith University, Nathan, QLD 4111 Australia; 3 School of Environment and Science (ESC), Griffith University, Nathan, QLD 4111 Australia

**Keywords:** Field effect transistor, bioFET, Wearable device, Biosensor, Non-invasive monitoring, Sweat, Tear, Saliva, Interstitial fluid

## Abstract

The rapid advancement of wearable biosensors has revolutionized healthcare monitoring by screening in a non-invasive and continuous manner. Among various sensing techniques, field-effect transistor (FET)-based wearable biosensors attract increasing attention due to their advantages such as label-free detection, fast response, easy operation, and capability of integration. This review explores the innovative developments and applications of FET-based wearable biosensors for healthcare monitoring. Beginning with an introduction to the significance of wearable biosensors, the paper gives an overview of structural and operational principles of FETs, providing insights into their diverse classifications. Next, the paper discusses the fabrication methods, semiconductor surface modification techniques and gate surface functionalization strategies. This background lays the foundation for exploring specific FET-based biosensor designs, including enzyme, antibody and nanobody, aptamer, as well as ion-sensitive membrane sensors. Subsequently, the paper investigates the incorporation of FET-based biosensors in monitoring biomarkers present in physiological fluids such as sweat, tears, saliva, and skin interstitial fluid (ISF). Finally, we address challenges, technical issues, and opportunities related to FET-based biosensor applications. This comprehensive review underscores the transformative potential of FET-based wearable biosensors in healthcare monitoring. By offering a multidimensional perspective on device design, fabrication, functionalization and applications, this paper aims to serve as a valuable resource for researchers in the field of biosensing technology and personalized healthcare.

## Introduction

The popularity of wearable biosensors has been increasing with the development of smartphones and other mobile devices, offering the remarkable capability of continuous and real-time collection of physiological data, thereby providing valuable insights into individuals’ performance and health [1, 2]. These biosensors, which incorporate biological recognition elements into their design, hold great potential in managing chronic conditions and supporting remote monitoring. This capability was not possible before with traditional analytical methods. Despite their high sensitivity and complexity, lab-based approaches often lack real-time and point-of-care capabilities. For instance, mass spectrometry [3, 4] can detect a wide range of biomarkers simultaneously. However, its reliance on laboratories, trained technicians, high cost, and time-consuming procedures makes it impractical from a decentralized clinical perspective. Similarly, ELISA [5, 6], which is extensively used in laboratory environments for clinical diagnosis of biochemical species, suffers from lengthy analysis time, limited usability outside traditional diagnostic laboratories, reliance on bulky analytical instruments [7], and the requirement for larger sample sizes [8].

**Fig. 1:**
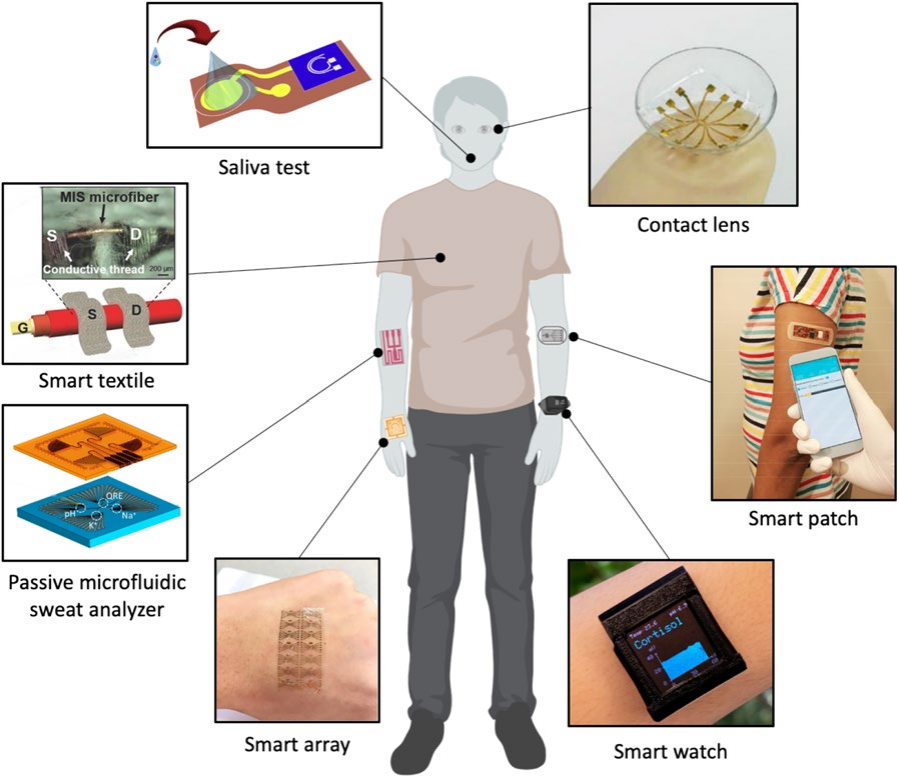
Wearable FET biosensors for diagnostics and health monitoring applications. Clockwise from top: Contact-lens biosensor (reprinted with permission from Ref. [16] Copyright 2019 Wiley); Smart patch biosensor (reprinted with permission from Ref. [1] Copyright 2019 American Chemical Society); Smart Watch biosensor (reprinted with permission from Ref. [17] Copyright 2022 Science); Smart array biosensor (reprinted with permission from Ref. [18] Copyright 2020 Elsevier); Passive Microfludic Sweat Analyzer biosensor (reprinted with permission from Ref. [19] Copyright 2018 American Chemical Society); Smart textile biosensor (reprinted with permission from Ref. [20] Copyright 2016 Wiley); Saliva test biosensor (reprinted with permission from Ref. [21] Copyright 2019 Elsevier)

To date, considerable efforts have been dedicated to the development of next-generation wearable biosensors. Early studies focusing on the non-invasive and dynamic measurement of biomarkers available in biofluids (e.g. interstitial fluid, saliva, tear, sweat). These wearable biosensors have been demonstrated for analytes collected from head-to-toe application sites, Fig. 1 [9]. Approaches for detecting biomolecules are for isntance electrochemical, micro-cantilever, fluorescence, colorimetric, chemoresistive and surface plasmon resonance (SPR) techniques, which have their respective advantages and disadvantages [10, 11]. These sensors offer high specificity and can detect substances at low concentration, making them suitable for early detection and real-time analysis. Electrochemical based immunosensors, including amperometric, impedimetric and potentiometric, have demonstrated novel and unique detection platform [12, 13]. Among these sensors, FETs have gained significant interest due to their advantages such as quick sample screening, label-free detection, wide dynamic range, and cost-effective fabrication processes, particularly on flexible substrates surpassing the capabilities of existing methods [14, 15].

Classical field effect transistors (FET) have been the backbone of modern electronic devices with gate-controlled current flowing through semiconducting channels. State-of-the-art micro/nanofabrication technologies make it possible to jam pack billions of transistors in a chip with size smaller than a human finger. This degree of miniaturization has led to a substantial increase in processing power, but at the same time a reduction in power consumption, and production cost. A few outstanding reviews exist on wearable FET sensors in different aspects. Li et al. [22] reviewed the recent advances in wearable devices based on flexible field-effect transistors including sensors for pressure, temperature, chemical, and biological analytes. Chen et al. [23] provided critical evaluation on multidisciplinary technical details, including sensing mechanism in detecting biomolecules, response signal type, sensing performance optimization, and the integration strategy. Dai et al. [24] review the recent advances of field-effect transistor sensors based on 2D materials, from the material, operating principles, fabrication technologies, proof-of-concept applications, and prototypes, to the challenges and opportunities for their commercialization.

The present review provides a comprehensive overview on FET-based wearable biosensors from various perspectives including sensing mechanisms, classification of device according to gating mode, materials, geometry, recognition elements and sampling. Next, we describe fabrication and surface modification techniques of FET biosensors. Furthermore, we outline the various principles of probe that can selectively detect the specific biological elements in terms of enzyme, antibody/nanobody, aptamer and ion-selective membrane. ﻿Additionally, we discuss physiological relevance of monitoring key biomarkers with wearable biosensors. Finally, we critically review and discuss challenges that greatly affect the future development of wearable FET biosensor.

### Structure and working principle of FETs

**Fig. 2:**
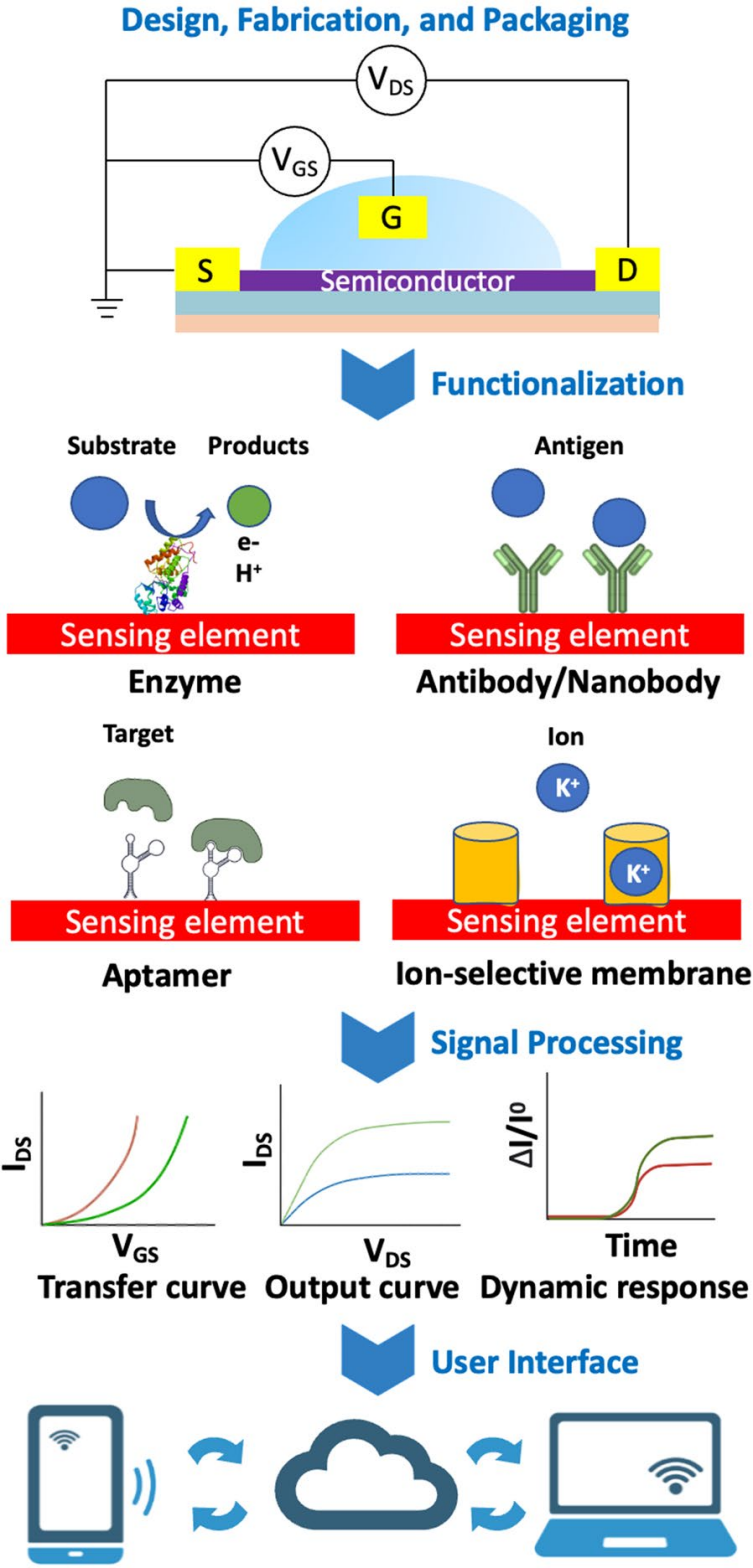
Illustration of a biological and chemical FET sensor platform. From top to bottom: Devices are fabricated and packaged by micromachining processes; Sensing surfaces are functionalized with probes to capture biomarkers; Charged biomarkers causes potential changes in sensing channels, then various electrical characteristics are measured; Analog signals are collected, transformed to digitals then logged to "cloud” data services for remote accesses

Field-effect transistor-based sensors (FET) are analytical devices that can selectively detect the concentration of a biological molecules. FET sensors typically comprise: a dielectric insulating layer, a semiconductor layer, and three electrodes (drain, source, and gate), Fig. 2.

The flow of current (I_DS_) between drain and source electrodes of a FET is regulated by a variable voltage (V_GS_) applied between the gate and source electrodes [22]. This applied voltage prompts a redistribution of the electric field within the dielectric layer, leading to the creation of a dual-electric layer. Consequently, charge carriers can move through the semiconductor layer close to the interface adjacent to the dielectric layer [25, 26]. The conductive behaviors displayed by these charge carriers vary depending on the relationship in energy levels between the semiconductor and the drain/source electrodes. More specifically, either holes (h^+^) or electrons (e^−^) can act as charge carriers in the semiconductor layer. In an n-type FET, when a positive voltage is applied to the gate, a channel is generated, allowing electrons to travel from the source to the drain, i.e., conduction [27]. Conversely, if a negative gate voltage is applied, the n-type channel is sealed off, preventing conduction by the carriers. With a p-type FET, the scenario unfolds in reverse, wherein a positive (negative) gate voltage deactivates (activates) the transistor.

Charged molecules that bind to the active layer, whether that be a gate electrode or a semiconductor channel, can cause the charges within the semiconductor material to redistribute, thereby modifying the conductance of the FET channel. When the target analyte interacts with the functionalized sensing layer of the FET device—typically either a semiconducting or a dielectric layer—it alters the electrical characteristics of this active layer at the molecular level. Consequently, the distribution of charge carriers within this layer changes, resulting in fluctuations in the output current of the FET. These fluctuations which can be measured as electrical signals, can either indicate the presence of the target analyte or changes of its concentration [22].

The Debye screening length, also called Debye length (λ_D_), is a physical distance where the charged analyte is electrically screened by the ions in the solution, strongly influences the sensitivity of immunosensor or electrochemical devices in high ionic strength media. The Debye length (λ_D_) in an electrolyte is given as [28]:

$$\lambda_{D} \, = \,\sqrt {\frac{{\varepsilon_{0} \varepsilon_{r} K_{b} T}}{{2N_{A} q^{2} I}}} ,$$

Where ε_0_ corresponds to the vacuum permittivity; ε_r_ is the relative permittivity of the medium; *k*_B_ is the Boltzmann constant; *T* is the absolute temperature; *N*_A_ is the Avogadro number; *q* is the charge on an electron; and *I* is the ionic strength of the solution. According to this Debye theory, an increase in ion concentration reduces the Debye length due to charge screening by counter-ions, lowering the sensitivity of the device.

### Classification of device

**Fig. 3:**
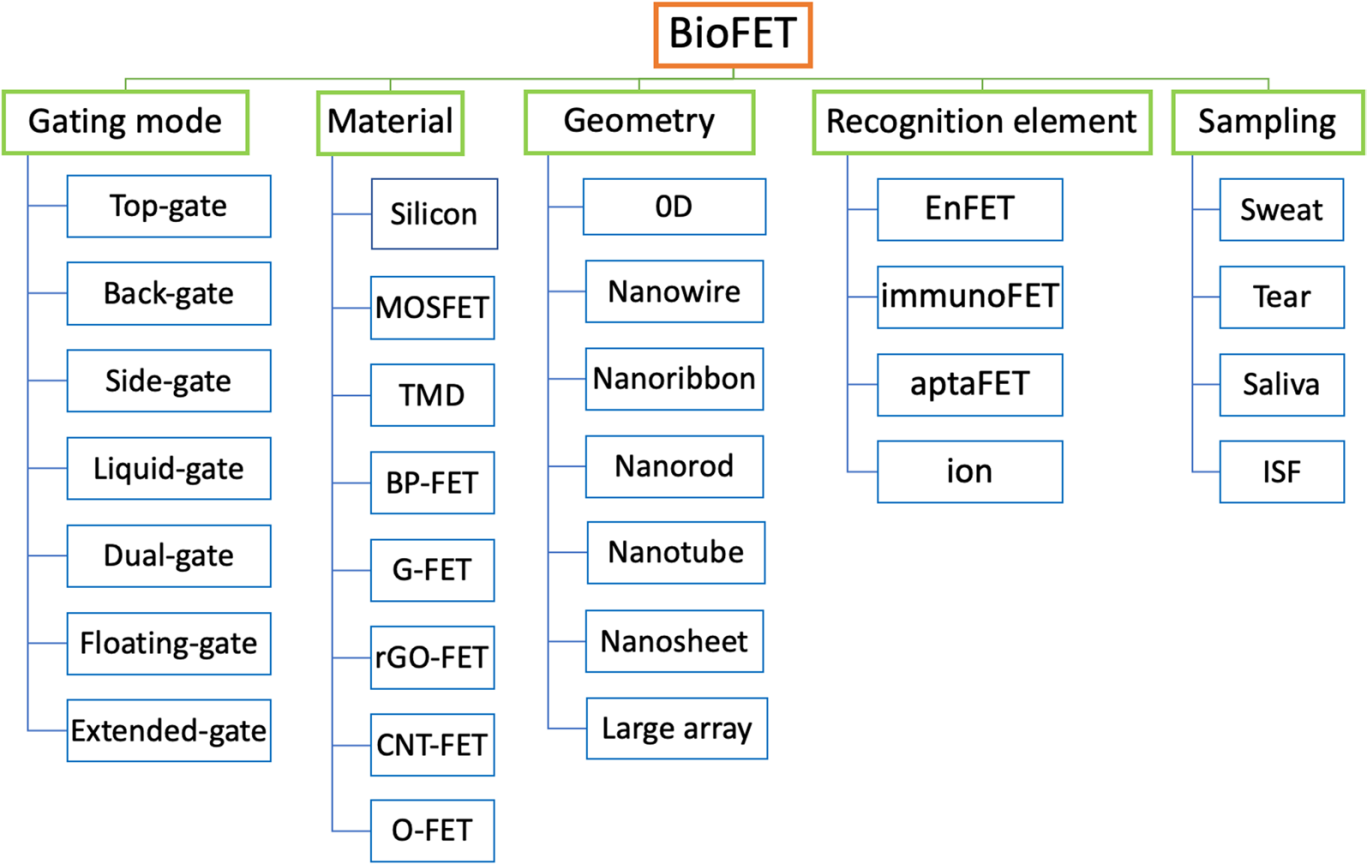
Representative classification of bioFET sensors for wearable devices

Figure 3 demonstrates different classification systems for FET biosensors according to architecture, material, geometry, recognition and sampling.

#### Architecture

**Fig. 4:**
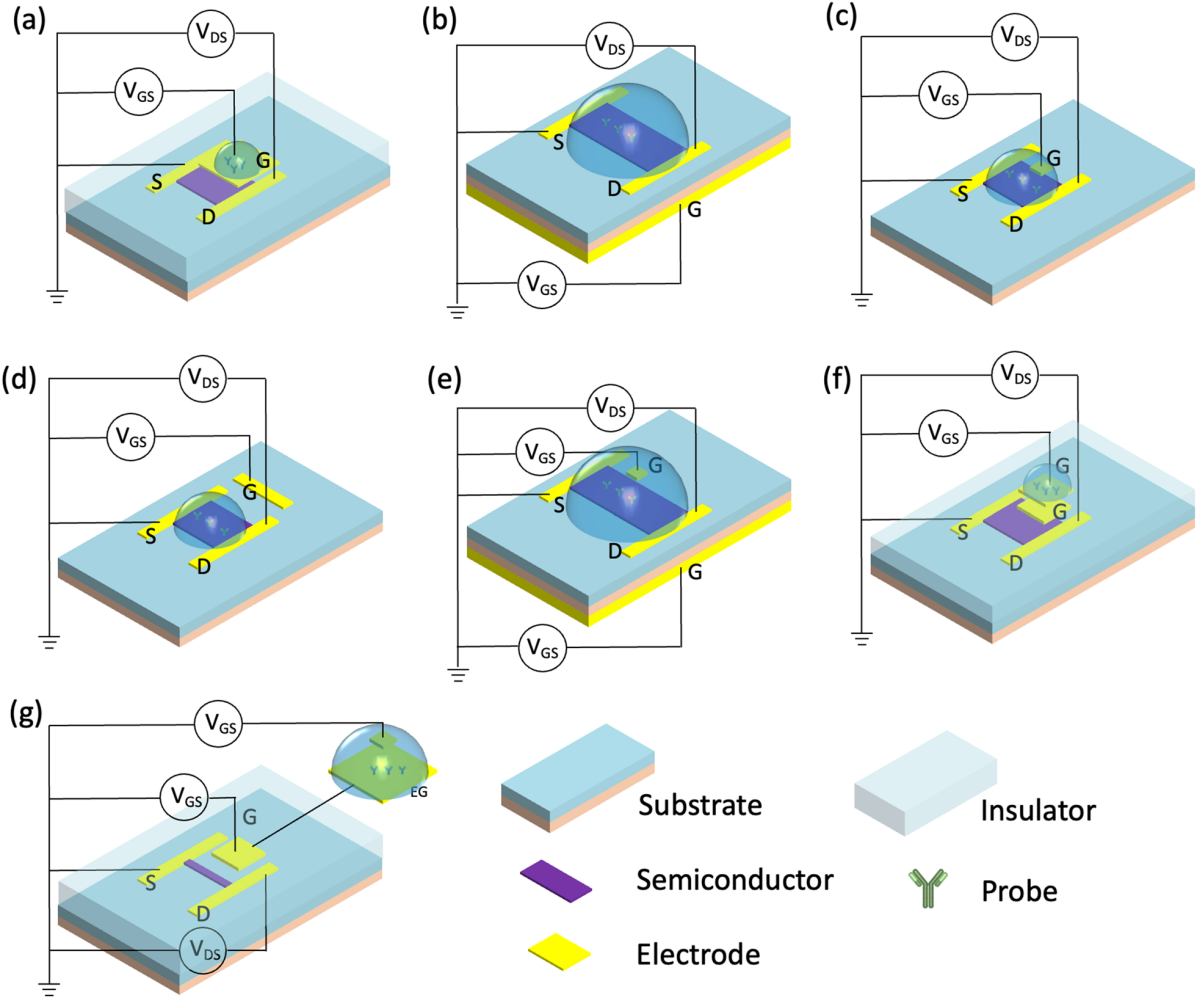
Design blocks of FET sensors. Representative FET structures with schematic illustration of the **a** top-gate, **b** back-gate, **c** side-gate, **d** liquid-gate, **e** dual back-gate and liquid-gate, **f** floating-gate **g** extended-gate

This classification method divides the gate architecture of FET biosensors into single gating (top-gate, back-gate, liquid-gate, side-gate) or complex-gating (dual-gate, floating-gate, extended-gate), Fig. 4. Additionally, FETs can be divided into top-contact and bottom-contact types according to the contact positions of the semiconductor and source/drain electrodes. Source/drain electrodes are deposited on an insulating layer in the top-contact type, whereas the source/drain electrodes are positioned above semiconductor layers in the bottom-contact type [29]. Generally, top-contact structures have a lower contact resistance and a higher mobility due to the technology used to fabricate the sensors. However, the bottom-contact types tend to have shorter channels.

Typically, top-gate biosensors are effective detectors as the top gate can also be used as the sensing component instead of semiconductor channel, Fig. 4a. Additionally, producing top-gate biosensors is relatively simple, requiring only contact lithography patterning and metal lift-off technology during the fabrication process [30]. For instance, Chu et al. [31] developed an electric-double-layer FET that can directly detect various proteins in physiological high ionic strength solutions. The gold top gate in this device is separated from the active channel. The probe is immobilized on the gate, resulting in sensitive detection of the analyte with a concentration as low as 1 fM.

Compared to top-gate configuration, back-gate biosensors often have a larger sensing area, Fig. 4b. In this type of sensor, the silicon substrate is commonly employed as the back gate, and silicon dioxide serves as the dielectric layer for the gate [30]. Guo et al*.* [32] developed a MoS_2_ FET, employing a basic back-gate configuration directly fabricated onto a SiO_2_/Si substrate through the standard nanofabrication process. This high-performance MoS_2_ transistor is large in area and ultrathin, making it relatively easy to integrate into a soft, smart contact lens where photodetectors, glucose sensors, and temperature sensors monitor the tear fluid.

The liquid-gate (also called solution-gate) is the most common type of FET as it presents the best simulation of the human physiological environment for binding biomolecules, Fig. 4c. When the FET operates in an electrolyte solution, reference electrodes are immersed in a solution, which provides bias voltage through a liquid gate. An electric field establishes at the interface between the electrolytes and the semiconductor. As a result, an electric double layer (EDL) forms, and modulates the potential and conductivity of the channel [24]. This EDL at the semiconductor interface not only makes the devices highly sensitive to a range of analytes, but it also allows for operation at low gate potentials. Wang et al. [17] developed a wearable liquid gate In_2_O_3_-FET, which relies on aptamers to measure cortisol level in sweat. An Ag/AgCl reference electrode on the chip is fabricated simply by depositing Ag/AgCl ink, which supports linear gate-source sweep voltage biasing.

Another FET architecture is side gate (or co-planar gate). In this type, the gate is placed in the same plan as the channel layer (Fig. 4d). In this case, the side gate can simultaneously bias several nearby semiconductor channels for multi-functional biosensing. For instance, simultaneous monitoring of temperature, pH, and neurotransmitters (dopamine and serotonin) was achieved in a multiplexed platform using this architecture [33].

In a dual-gate FET, two insulated gates (bottom-gate and top-gate electrodes, or bottom-gate and liquid gate electrodes) offer two different V_GS_ for independent modulation, Fig. 4e. This configuration increases the sensor’s response, improves signal-to-noise ratio, and reduces signal drift and hysteresis [34]. Capua et al. [35] developed a SiNW FET using this configuration to detect C-reactive protein. The sensor has excellent stability, low hysteresis, great sensitivity, and a negligible shift over time. These properties are highly advantageous in applications, where the biomarkers in body fluids need to be continuously monitored.

Floating-gate type FET sensors are designed to work in solutions, and are a relatively recent development. An additional metal floating control gate electrode is electrically isolated between the original top-most gate (now called the control gate) and the channel, creating a floating node in direct current. This floating node can store the charge and control the channel’s conductivity, Fig. 4f. An oxide layer surrounding the floating gate keeps the electrons trapped such that the device can store an electric charge for an extended period of time without needing to connect to a power supply. Liang et al. developed a wafer-scale uniform floating-gate carbon nanotube FET system [36]. An ultrathin Y_2_O_3_ high-κ dielectric layer in the floating-gate structure increases the sensitivity and amplifies the response of the FET, as compared to counterparts without a Y_2_O_3_ layer. The improvement is attributed to a dominant chemical gate-coupling effect in the response mechanism of the sensor [36]. The theoretical LOD is as low as 6 particles/mL.

Extended-gate FET (EG-FET) expands the gate electrode off-chip to enforce separate wet and dry environments, Fig. 4g. Only the detector portion is immersed into the solution, while the transducer remains in a completely dry environment. The sensor electrode is linked to a MOSFET gate and elongated with a metal signal line. The advantage of this setup is that the majority of the electrical signals is isolated from the measurement environment, making it a straightforward and dependable encapsulation approach. This configuration also minimizes environmental interference, including light-induced drift [37]. Fabricating this device is also substantially simple, allowing post-processing steps. In this category, Yang et al. [38] developed a low-cost and flexible ITO/PET EG-FET with roll-to-roll fabrication. The extended gate in this device has a simple enzyme functionalization for the detection of urea.

#### Semiconductor materials

Various semiconductor materials have been used to fabricate FET, including silicon [35], metal oxides [18], III-V materials [39], transition metal dichalcogenides [39], organic semiconductors [40], graphene [2], carbon nanotubes [41], and black phosphorous [42]. Several criteria need to be considered for selecting the semiconductor for a bioFET sensor. These include the electrical and mechanical stability of the measurement environment, the sensitivity of the material to light and temperature, the availability of the material in commercial quantities, and the compatibility is with large-scale fabrication on flexible substrates [43]. Table 1 provides a summary of the potential impact of material properties on biosensors.

**Table 1:** Impact of material property. Reprinted with permission from [43]

Consideration parameter	Potential impact
Materials bandgap, charge density and carrier mobility	Device performance and packaging requirements
Stability in ambient environment	Packaging, fabrication. e.g. A device passivation step may be necessary to protect the semiconductor material if it is unstable in ambient environments
Stability in the intended measuring condition	Device and signal reliability. e.g. In wet chemical and biological sensing, the material must be stable in the intended solution (e.g., pH, ionic strength)
Light and temperature sensitivity	Packaging and operational condition. e.g. Materials that are sensitive to visible light require much more stringent packaging and/or operation in the dark might be required. This requirement compromises the robustness of the device. High bandgap materials are generally less light and temperature sensitive
Compatible fabrication methods and their compatibility for flexible substrates	Dictates the fabrication route to be taken, how scalable production is, and decisions on the substrate to be used

FET-based wearable devices have been successfully applied to materials such as graphene, carbon nanotubes, silicon, In_2_O_3_, MoS_2_, ZnO, zinc titanium oxide, and organic semiconductors. These materials are compatible with substrates such as Si/SiO_2_, polyimide, polyester PET, poly(dimethylsiloxane) (PDMS), polyethylenenaphthalate (PEN). When selecting a substrate, one must consider the processing temperature involved in the device fabrication, along with other physical properties of the substrate such as the glass transition temperature, flexural modulus, Young’s modulus, optical transparency, thermal expansion, and adhesion to active materials.

#### Semiconductor geometry

Nanoscale semiconductor materials have typically a thickness of a single atom or perhaps a few atoms. Measuring less than 5 nm deep, their lateral size can range from sub-micrometers to centimeters [24]. Table 2 summarizes the strengths and weaknesses of some of the common nanoscale materials.

**Table 2:** Common geometries of nanoscale semiconducting materials

Geometry	Comment	Examples
Nanowire	• One-dimensional structure with a high aspect ratio (length much larger than diameter) • Good electrostatic control and gate control due to its small dimensions • Difficult to fabricate at exact sizes	[35]
Nanoribbon	• Two-dimensional structure with a rectangular shape and a high aspect ratio • Allows for the precise control of electronic band structures and properties • Comparable sensitivity to nanowire but fabrication is easier	[33]
Nanotube	• One-dimensional cylindrical structure with a tubular shape • Exhibits excellent electrical properties, such as high carrier mobility and current-carrying capacity • Surface modifications are difficult	[41]
Nanorod	• One-dimensional structure with a rod-like shape, typically elongated in one direction • Provides a large surface-to-volume ratio, enabling efficient charge transport and enhanced device performance	[44]
Nanosheet	• Two-dimensional structure with an atomically thin and planar geometry • Precise control over thickness and composition • Excellent electrical conductivity (High I^on^/I^off^ ratio) and mechanical flexibility	[16]

#### Other classifications

Another way to categorize FET sensors is based on the substance used to recognize the analyte. For example, if a FET is designed to detect an analyte through an enzymatic reaction, the sensor is called an enzyme-FET or an EnFET. Other categories include ISFETs, aptaFETs, immunological FETs, and DNA-FETs. For wearable devices, several recognition elements also known as probes have been used to monitor biomarkers in body fluids. Finally, wearable FET device can be classified based on the biofluid samples, such as sweat, tear, saliva, ISF. Further details about recognition element and samples will be discussed in other sections of this paper.

### Fabrication of semiconductor materials

**Fig. 5:**
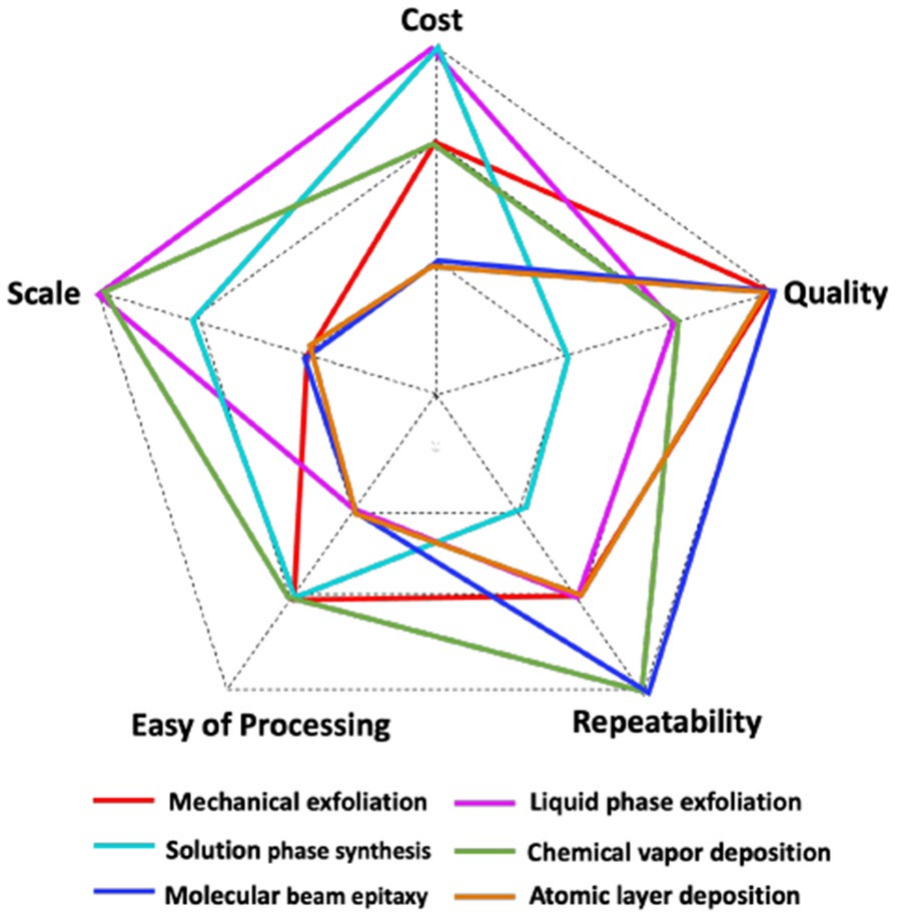
Qualitative comparison of common fabrication techniques of thin films. Repaint with permission from Ref. [24]. Copyright 2022 American Chemical Society

The main function of semiconductors in FET sensors is to facilitate the flow of current, which critically impacts the sensitivity [43]. Various approaches can be employed to fabricate FET sensors, including top-down machining techniques, bottom-up synthesis techniques, and assembly strategies. Figure 5 summarized common fabrication techniques of thin films along five qualitative parameters, including cost, quality, repeatability, scale, and ease of processing [24].

The top-down approach is a process of breaking down the bulk materials into smaller, micro and nanoscale structures. For silicon nanowire (SiNW)-FET sensors, top-down machining is well established with advanced lithography and microfabrication techniques to make microdevices in batch in a controlled setting, allowing both cost-effective and easily scalable to produce large quantities [45]. Commencing with a silicon-on-isolator (SOI) substrate, the SiNW sensor structure is designated using pattern transfer methodologies, including electron-beam lithography, nanoimprint lithography, or sidewall transfer lithography. The subsequent etching process encompasses reactive ion etching, wet chemical etching, or a hybrid approach integrating both techniques, facilitating the transfer of the designed structure onto the uppermost silicon layer of the SOI substrate. Further microfabrication techniques are then employed to finalize the devices with source drain, ohmic contact establishment, gate dielectric layer implementation, and passivation layer. For top-down machining of materials other than silicon, mechanical/liquid exfoliation is a common technique. Mechanical exfoliation is reported to produce exceptional quality with minimal defects by peeling-off atomically thin layers from a bulk material [46, 47]. Graphene, the first 2D materials discovered in a laboratory, was prepared by this method [48], simply with adhesive tapes. However, the process is time-consuming and labour-intensive, especially when aiming to obtain large area of thin flakes [45]. It is also hard to get uniform samples as there are lots of flakes with different number of layers randomly dispersed on the substrates [49]. Mechanical exfoliation is one of the most used top-down techniques for producing metal oxide nanosheets with high quality and degree of crystallinity [50]. Recently, Zhang et al. reported a novel mechanical exfoliation process to prepare MoS_2_ nanoflakes with thermal treatment to improve the size and yield of material [51]. Liquid phase exfoliation, on the other hand, offers an alternative replacement to isolate and disperse thin layers of nanomaterials from their bulk crystals in a liquid medium. The technique involves breaking down the bulk crystals of the material into thinner layers by applying mechanical or ultrasonic forces. The resulting nanosheets or nanoparticles can be easily dispersed in a liquid solvent to form a stable colloidal suspension [52]. Liquid exfoliation typically produces materials with high yield, good quality and rather low cost [49]. This technique has been applied to a wide range of material including carbon nanotube [53], graphene [54, 55], and black phosphorus [56, 57].

Bottom-up synthesis involves assembling and growing materials from atomic or molecular precursors to form desired structures. Following this approach, SiNWs were vertically grown on a silicon substrate using various methods such as chemical vapor deposition (CVD) [58], oxide assisted growth [59] or metal-assisted chemical etching [60]. In the case of metal oxides, various structural forms such as nanoribbons, nanowires, nanorods, nanobelts, and nano thin films, can be created using vapor-phased techniques, which include depositing chemical vapors and physical vapors [43]. While CVD synthesizes nanostructures through chemical reactions in the vapor phase with the assistance of a noble metal catalyst. In contrast, physical vapor deposition (PVD) produces nanostructures by either thermal evaporation or plasma. CVD is also especially useful for transition metal dichalcogenides such as MoS_2_ [61], as large-scale materials can be grown on different substrates by CVD, and the materials can be easily transferred to other substrates [62]. However, this method needs an accurate control of experimental conditions, so it is still too complicated and expensive for mass-production [49]. Molecular-beam epitaxy (MBE) is a physical vapor deposition technique that can grow thin films of single crystals from various materials, including gallium nitride [63], zinc oxide [64] and organic semiconductor [65]. This sophisticated technique offers atomic-level precision over the growth of crystalline materials in an ultra-high vacuum environment. However, MBE has high equipment and operation costs, and may face challenges in scaling up due to its relatively slow growth rate [66]. Similar to CVD, atomic layer deposition (ALD) is a thin film deposition technique relying on surface chemical reactions of gaseous precursors, but these surface reactions occur through self-saturating gas-surface reaction mechanisms [67]. ALD offers the capacity for layer-by-layer deposition with exceptional control over film thickness, outstanding uniformity, and unparallel conformal coverage on nanostructured surfaces [68], albeit at the expense of its high cost and slow deposition rate [69]. Solution-phase synthesis routes, such as sol–gel and hydrothermal synthesis, can be employed to produce many metal oxide nanostructures and thin films [43]. These solution-based methods provide a cost-effective approach to create a diverse array of metal oxide nanostructures with controllable properties [29].

Assembly strategies are used to arrange and position nanomaterials onto specific locations of the FET sensor. For example, the floating-coffee-ring driven assembly takes advantage of the self-assembly behavior of nanoparticles in evaporating liquid droplets, resulting in a ring-like pattern of the deposited nanoparticles [70]. Dynamic-template-assisted meniscus-guided coating is another assembly strategy where the controlled motion of a template or meniscus is used to guide the deposition of materials onto desired areas of the sensor surface [71, 72].

These fabrication techniques contribute to the versatility and functionality of FET sensors, allowing for tailored sensor design and performance optimization in various applications. It is important to note that these techniques are not exhaustive. Advancements in nanotechnology and material science continue to expand the repertoire of fabrication methods for FET sensors.

### Functionalization techniques of semiconductor surface

Surface functionalization or immobilization involves attaching biological receptors onto a matrix or support, either on the surface or within it. This attachment can occur through physical or chemical means, through direct or indirect techniques, as long as ultimately the bioreceptors are coupled to the active layer of the sensor [73].

Regardless of the specific method used for immobilization, the technique should be straightforward to perform, highly reproducible, effective at preventing non-specific bindings, and robust to extreme environmental conditions [74]. Additionally, following the immobilization, the biomolecules should remain both easily accessible and chemically inert to the host structure. This will ensure the biosensor remains both functional and stable over time.

#### Physical adsorption

**Fig. 6:**
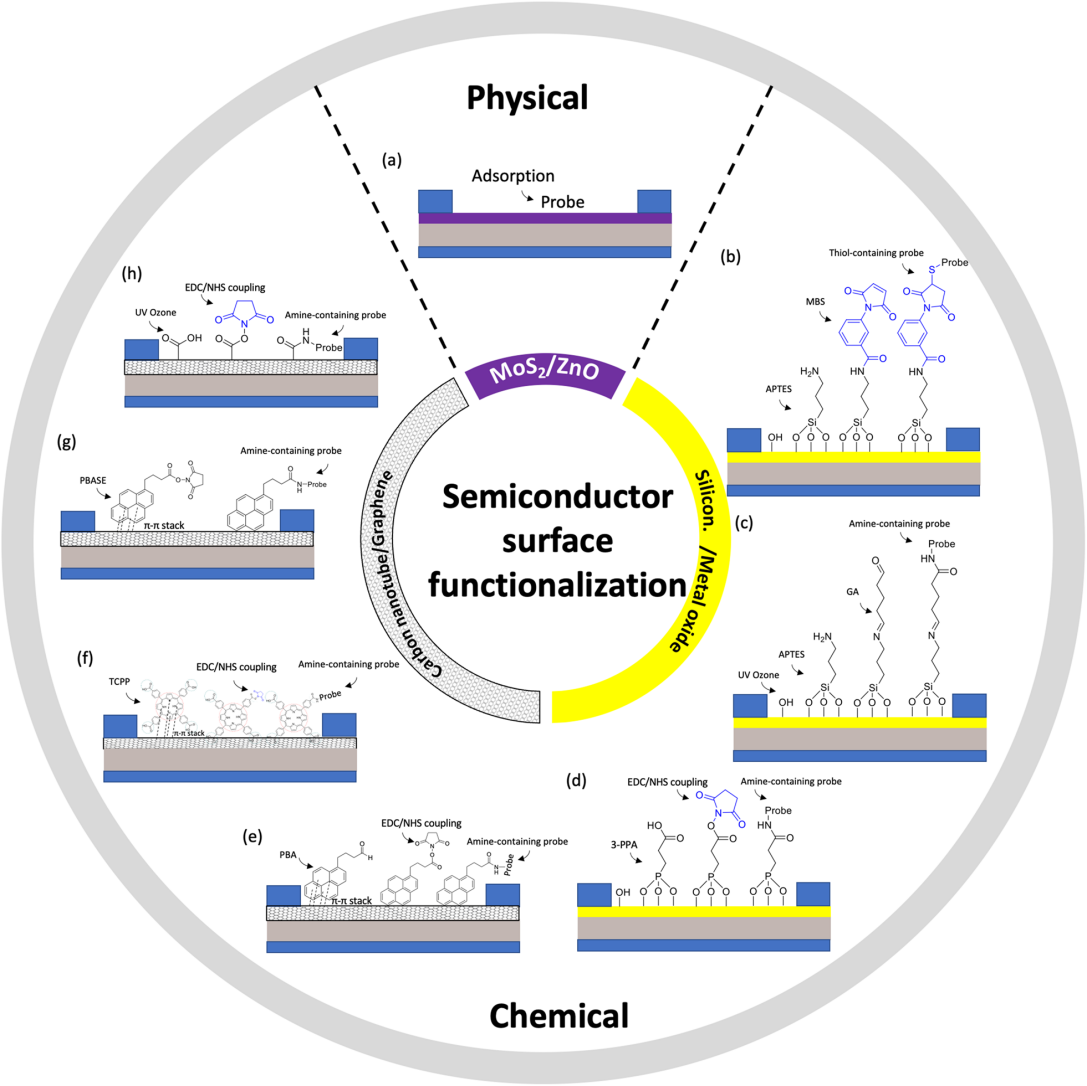
Surface functionalization/immobilization techniques on semiconductor

The simplest method of immobilizing biomolecules on a surface is physical adsorption (physisorption). This process involves attaching the biomolecules to the surface using weak and noncovalent binding or deposition forces, such as electrostatic interactions, hydrophobic interactions, van der Waals forces, and hydrogen bonding interactions between the sensor surface and the target analyte (Fig. 6a) [75]. In the physisorption process, biomolecules are typically immobilized on an electrode/semiconductor surface by immersing the surface into a solution containing the biomolecules and allowing them to bind to the surface during a fixed incubation period. A minimum bulk receptor concentration of 1 µM is required for a typical incubation time of 1 h to guarantee maximum surface coverage [76]. Subsequently, the surface is washed with a buffer solution to remove any unbound biomolecules [75].

However, there are certain limitations associated with physisorption. Due to the weak and noncovalent nature of the interactions, the performance, stability, and reusability of the sensor can be significantly affected by factors such as temperature, pH, concentration, and ionic strength. As a result, this method has not received extensive attention. One major drawback of immobilization through physisorption is the possibility of desorption of the bioreceptors from the surface during measurements, leading to decreased sensitivity. Additionally, non-specific adsorption of interfering molecules on the sensor surface is another issue that can negatively impact the sensor's accuracy and specificity [75].

In particular, Guo et al. [32] fabricated a MoS_2_ FET immobilized with glucose oxidase via physical adsorption. The measured glucose concentration in phosphate buffer was from 0.1 mM to 0.6 mM, which is within the typical range of human tear’s glucose level. Similarly, Zong et al. [44] developed a glucose sensor with ZnO FET. The FET sensor is fabricated via hydrothermal growth of semiconducting ZnO nanorods between source and drain microelectrodes. Following the fabrication process, the semiconductor was incubated with glucose oxidase solution overnight to maximize the adsorption. This approach was able to detect glucose with an LOD of 1 μM.

#### Chemical modification

Chemical approaches offer a solution to address the limitations of physisorption and generally achieve better performance. These approaches involve creating a strong and stable attachment between biomolecules and the electrode surface through covalent bonding, crosslinking, and bioconjugation affinity using the functional groups present on the surface [73].

In the case of FET sensors, the surface is functionalized with a chemical agent that uses covalent bonds to immobilize specific bioreceptors. Covalent immobilization provides a stable and permanent attachment of the bioreceptor to the FET surface, enhancing the device’s sensitivity and specificity. This approach ensures a reliable and efficient biosensing system capable of accurately detecting the target analyte [43].

Prior to functionalization, the semiconductor surface should be activated with either UV-ozone, oxygen plasma [38] or an ammonia/hydrogen peroxide mixture. These treatments create − OH or − COOH groups on the surface, making it more receptive to a reaction with any organosilanes during the subsequent functionalization step.

When it comes to metal oxide semiconductors, organo-silanization is a commonly used technique for immobilizing biomolecules on the surface of oxides such as SiO_2_ and various forms of glass. This process involves functionalizing the oxide surface with specific organosilane compounds. Two frequently used alkoxysilanes for this purpose are 3-aminopropyl-triethoxysilane (APTES) and 3-glycidyloxypropyltrimethoxysilane (GOPTS). Other alkoxysilanes like 3-mercaptopropyltrimethoxysilane and 3-trimethoxysilyl propyl aldehyde may also be used. Following APTES functionalization, the amine functional groups can be further reacted with a linker such as m-Maleimidobenzoyl-N-hydroxysuccinimide ester (MBS, Fig. 6b) or glutaraldehyde (GA, Fig. 6c). These linkers introduce functional groups that can be covalently bound to various molecular thiol- or amine- containing probes, such as nucleotide probes, aptamers, antibodies, nanobodies, proteins, or enzymes. For instance, Liu et al. [33] functionalized In_2_O_3_ FET platform with thiolated aptamer using APTES and MBS linker. The platform was able to simultaneously detect neurotransmitters, such as serotonin and dopamine, in real-time with a detection range from 10 fM to 1 μM. Similarly, Hayashi et al. [77] used APTES and GA linker to immobilize Jacalin into the surface of SiO_2_ bioFET. The device could specifically detect secretory immunoglobulin A in sweat at concentrations ranging from 0.1 μg/mL to 100 μg/mL. These studies demonstrated the utility of FET biosensors in monitoring condition on mental health to prevent depression.

Approaches with phosphonate chemistry offer some advantages over organosilanes [43, 78]. First, they can be applied to a broader range of metal oxides compared to silane chemistry, making phosphonate chemistry applicable to a wider variety of surfaces. Secondly, phosphonic acid-based monolayers are less sensitive to moisture than organosilanes. This characteristic is important for practical use such as storage, because the stability of a monolayer is less affected by the humidity of the environment. Furthermore, phosphonate chemistry is less prone to self-condensation. In other words, phosphonic-acid-based reagents should minimize the amount of undesirable byproducts compared to organosilanes, leading to more predictable and controllable biofunctionalization processes [29]. In particular, Li et al. [79] submerged In_2_O_3_ nanowire into 3-phosphonopropionic acid, resulting in binding of the phosphonic acid into the surface of the semiconductor. Next, the carboxylic group was activated by carbodiimide chemistry, which served as the anchor for amine-containing probe such as monoclonal antibody (Fig. 6d). Real-time detection in solution has also been demonstrated for analyte down to 5 ng/mL.

In terms of carbon nanotube channel, the surface can be functionalized with 1-pyrenebutyric acid (PBA). PBA contains a pyrene group that can be attached to carbon nanotube via π-π* stacking, while the carboxyl group can be used to covalently anchor to amine-containing probe using carbodiimide chemistry, which typically involves EDC (1-ethyl-3-(3-dimethylaminopropyl)carbodiimide hydrochloride) /NHS (N-hydroxysulfosuccinimide) coupling reaction, Fig. 6e. During this coupling process, the EDC creates a reactive O-acylisourea ester, rendering the surface temporarily unstable. This O-acylisourea ester then reacts with the NHS to form an amine-reactive NHS ester, while the surface remains semi-stable. Next, amine group from probe reacts with the amine-reactive NHS ester to form a stable amide bond that immobilizes the probe onto the NHS present on the surface of carbon nanotubes. Filipiak et al. [80] employed this strategy to functionalize SWCNT-FET with nanobody receptor such that the device can detect antigen with high selectivity and sub-picomolar detection limit with a dynamic range exceeding 5 orders of magnitude. Long-term stability measurements reveal a low drift of SWCNTs of 0.05 mV/h, making it promising for continuous real-time monitoring of biomarkers.

Similarly, for graphene semiconductors, the central macrocycle of Tetrakis (4-carboxyphenyl) porphyrin (TCPP) can be attached to the graphene’s honeycomb carbon network via π-π* stacking. Subsequently, amine-containing probe could be immobilized via EDC/NHS coupling to the carboxyl groups surrounding the macrocycle of TCPP (Fig. 6f). Zhang et al. [81], for example, used this approach to functionalize a GFET with a cortisol aptamer for salivary cortisol tests. Notably, this TCPP decoration not only enhances the sensitivity of liquid gate-GFETs to cortisol but also shields the oxygen-containing groups, thereby reducing any response to pH variations in the samples [81].

Alternatively, a graphene channel can be biochemically functionalized by using 1-pyrenebutyric acid N-hydroxysuccinimide ester (PBSE). PBSE contains a pyrene group that interacts with graphene through π-π stacking, at the other end, it has an ester group that reacts with primary amines. Next, a probe containing NH_2_ can be directly linked to the PBSE by forming amide bonds without EDC/NHS chemistry (Fig. 6g). Zhuang Hao et al. [82] followed this approach to functionalize a GFET involving an aptamer to monitor insulin. Initially, the sensor was submerged in a PBSE solution. It was then washed with dimethylformamide (DMF) to eliminate any unbound PBSE. Next, the device was rinsed with PBS and exposed to an aptamer solution. After PBS rinsing, ethanolamine was applied to the graphene channel to deactivate and block any excess reactive groups left on the graphene surface. The authors, later on, continued with this procedure for the immobilization of IL-6 aptamer to monitor cytokine level in saliva [83].

Another technique for activating the surface of graphene channel with a carboxylate group is utilizing ultraviolet ozone (UVO). However, prolonged exposure to UVO can excessively degrade the graphene's resistance, so UVO exposure is typically limited to two minutes [2]. Once UVO has been applied, amine-containing probe can be immobilized through an EDC/NHS coupling reaction, Fig. 6h. In particular, Ku et al. [2] used this method to functionalize a GFET platform with cortisol monoclonal antibody. The platform was able to measure cortisol concentrations in real time with a detection limit of 10 pg/mL.

### Gate surface functionalization techniques

Using a gate as an active layer is another common approach for FETs. In some cases, the surface of the gate electrode is functionalized with probes.

**Fig. 7:**
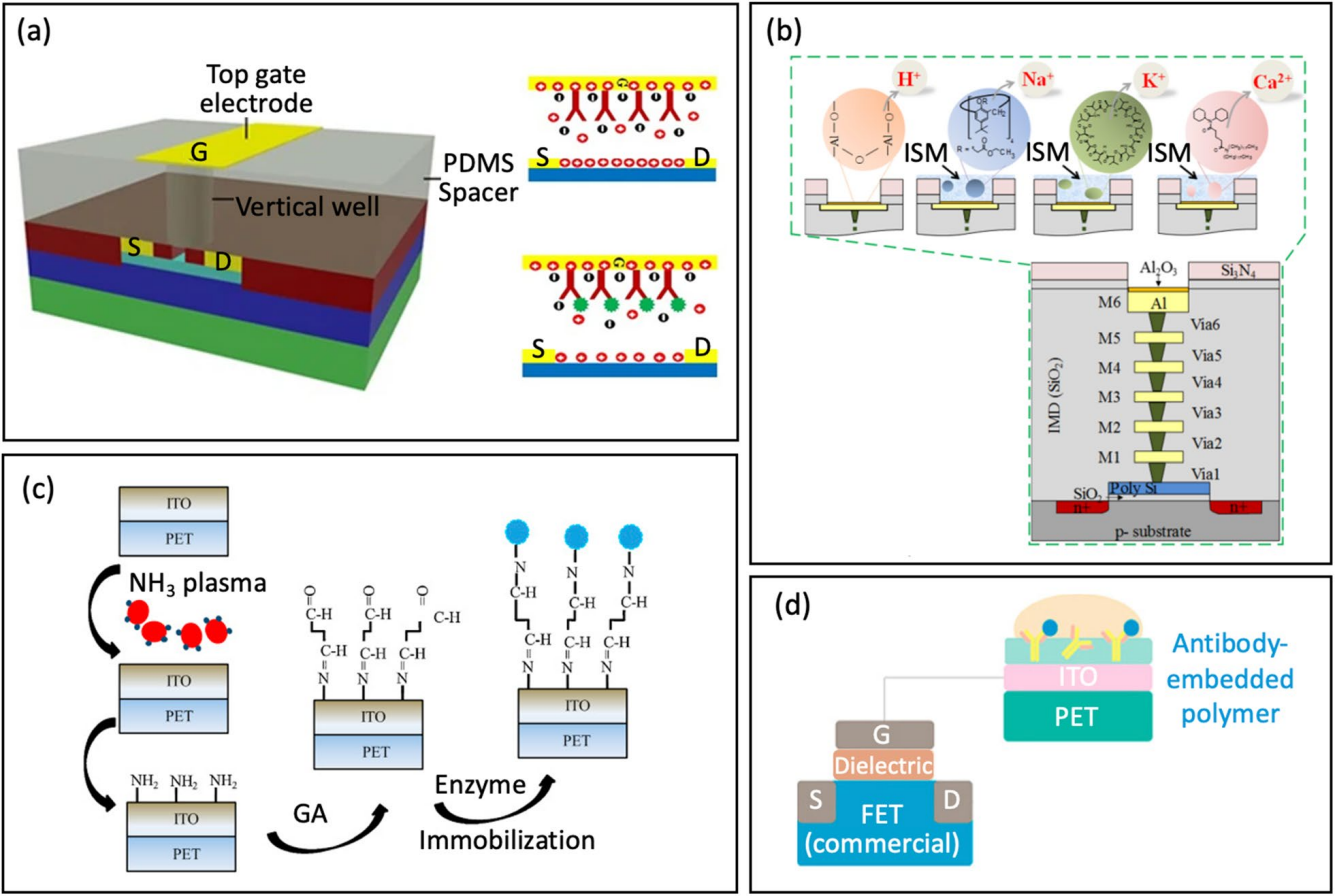
**a** Surface functionalization/immobilization on top-gate gold electrode with antibody via thiol-gold adsorption (reprinted with permission from Ref. [84] Copyright 2017 Springer Nature). **b** Cross-sectional view of the 3D-EMG-ISFET sensor functionalized with ion selective membranes sensing (reprinted with permission from Ref. [1] Copyright 2019 American Chemical Society). **c** Schematic of enzyme immobilization on indium tin oxide films on flexible polyethylene terephthalate substrates (reprinted with permission from Ref [38. Copyright 2013 Elsevier). **d** Antibody-Embedded polymer coupled to extended gate (reprinted with permission from Ref. [85] Copyright 2018 American Chemical Society)

For example, thiol-containing probes such as antibodies and aptamers can readily be used to functionalize top-gate gold electrodes through strong S–Au covalent binding to detect proteins [84], Fig. 7a. Proteins binding to these functionalized electrodes result in a drop in gate voltage, which redistributes the charge density locally around the gate. This induces a change in charge density of the active channel. More importantly, this detection method does not require any dilution or washing processes to reduce ionic strength, yet it remains highly sensitive. The entire detection process only takes about 5 min.

Zhang et al. [1] introduced a Three-Dimensional Electrode-Metal-Gate Ion-Sensitive FET (3D-EMG-ISFET) for monitoring electrolytes in sweat, Fig. 7b. An efficient functionalization material for electrolyte-sensing is the ion-selective membrane (ISM). ISM has specific embedded ion receptor, known as an ionophore, in a polyvinyl chloride-based membrane ((PVC)/bis(2-ethylhexyl) sebacate (DOS)). This membrane is drop-casted onto the top of the 3D-EMG-ISFET sensing dielectric. The ionophore in the membrane interacts poorly with interfering ions while selectively interacting with target ion. Through these interactions, a junction potential is created, which changes the gate bias of ISFETs and is directly correlated with the ion activity at the liquid-to-ISM interface. In this particular study, three types of membranes were deposited for sensing, resulting in the creation of 3D-EMG-(Na^+^, K^+^, Ca^2+^) sensitive FETs [1].

Yang et al. [38] developed indium tin oxide (ITO) films on flexible polyethylene terephthalate substrates using different NH_3_ plasma treatment conditions. These ITO films serve as sensing electrodes for extended-gate FETs, Fig. 7c. An NH_3_ plasma treatment was then introduced to create amine groups on the ITO sensing membranes; thus, offering a simpler alternative to the complex procedures of inducing covalent bonding. Following the NH_3_ plasma treatment, a glutaraldehyde solution and urease were dripped separately onto the ITO membrane for further functionalization.

An alternative approach involves embedding the probe within a polymer, which is then coupled to the extended gate. Along these lines, Jang et al. [85] used an antibody-embedded PSMA sensing gate to detect cortisol, Fig. 7d. Incorporating the receptor into the polymer structure helped to bind the cortisol molecules in proximity to the membrane-substrate interface, effectively overcoming the problems associated with the Debye length (λ_D_). The reported LOD was 1 pg/mL in phosphate buffer saline, where λ_D_ is 0.2 nm.

### Probe

#### Enzyme (EnFET)

Enzyme-modified FET operates based on enzymatic reaction, where the enzyme catalyzes the conversion of a substrate into its product. This enzymatic reaction occurs within the enzyme membrane, leading to the change in accumulated charged carriers on the gate surface, directly proportional to the amount of analyte present in the sample [86]. As a result, protons are either generated or consumed, coupled with a change in pH levels. This change in pH can be measured using a pH-electrode as a reference electrode [87]. Therefore, EnFET biosensors can quantify the presence of the target analyte by correlating the changes in pH with the concentration of the analyte.

**Fig. 8:**
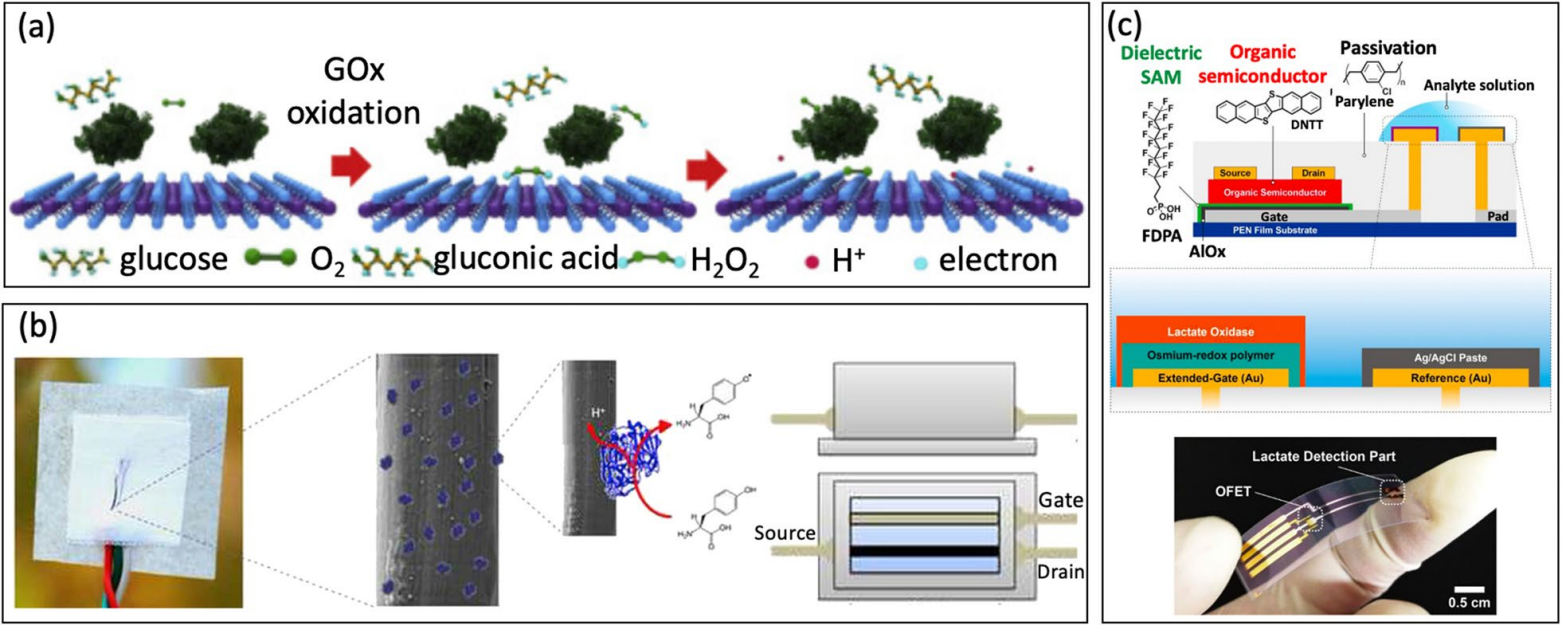
**a** Illustration of the sensing mechanism of the device with oxidation of glucose (reprinted with permission from Ref. [32] Copyright 2021 Elsevier). **b** Tyrosine sensing mechanism of organic electrochemical transistor functionalized with laccase (reprinted with permission from Ref. [94] Copyright 2017 Elsevier). **c** Lactate detection with extended-gate organic FET functionalized with lactate oxidase and osmium-redox polymer (reprinted with permission from Ref. [95] Copyright 2019 Springer Nature)

For instance, Guo et al. [32] used glucose oxidase (GOD) as the bio-enzyme for creating a MoS_2_ FET-based serpentine mesh sensor system to detect glucose on artificial eyeballs. To enhance glucose recognition and improve conductance, the team employed a large contact area with tear fluid to absorb and immobilize the enzyme. Initially, GOD (β-D-glucose oxidase from *Aspergillus niger*) is immobilized onto the surface of MoS_2_ [88, 89], Fig. 8a. The subsequent charge transfer mechanism occurs because the glucose is oxidized by the GOD to form H_2_O_2_, which then reacts with oxygen, generating hydrogen ions (H^+^) and electrons (e^−^):

$$D- Glucose + H2O2 + O2 \stackrel{GOD}{\to } D- gluconic acid + O2 + 2H+ + 2e-$$

This reaction generates free electrons, leading to an increase in device current due to the behavior of the n-type FET. GOD has a demonstrated high selectivity for glucose, with fully or quasi-reversible glucose conversion. Moreover, it is readily obtainable and has been proven to withstand conditions of extreme pH, ionic strength, and temperature [90–92]. This serpentine mesh sensor device can be placed directly onto the lenses for direct contact with tears, in contrast to conventional sensors and circuit chips embedded within the lens substrate. This design offered high detection sensitivity, mechanical robustness, and does not interfere with blinking or vision. Additionally, in-vitro cytotoxicity tests have shown good biocompatibility, making it a promising candidate for the next-generation of soft electronics in healthcare applications.

In a similar study, Kim et al. [93] developed a wearable contact lens capable of selectively and sensitively detecting glucose. To achieve this, the authors immobilized GOD on a graphene channel using a pyrene linker through π-π stacking interactions. GOD is attached to pyrene linker by forming an amide bond resulting from the nucleophilic substitution of N-hydroxysuccinimide. In their experiments, the authors demonstrated in-vivo glucose detection capability on a rabbit eye in real-time as well as wireless in-vitro monitoring of the intraocular pressure of a bovine eyeball. This innovative wearable contact lens holds significant potential for biomedical applications.

Moreover, Battista et al. [94] presented a textile wearable organic electrochemical transistor where the semiconducting polymer was fabricated on cotton fibers (the yarn). Recombinant fungal POXA1b laccase was immobilized by surface adsorption to detect Tyrosine (L-Tyr) (Fig. 8b). Lactate is a class of enzyme catalyzing the single-electron oxidation of phenolic compounds with associated four-electron reduction of oxygen to water. This direct electron transfer without mediator allows for the sensitive detection of Tyrosine in aqueous solutions. This approach allows for detecting Lactate with an LOD of 10 nM, making it a promising extensive utilization in sports, healthcare, and working safety.

Similarly, Minamiki et al. [95] developed an organic field-effect transistors using extended-gate electrode modified with a lactate oxidase on an osmium-redox polymer [69], Fig. 8c. The biosensing capability of the OFET is investigated through real-time measurement of lactate concentration in an aqueous environment. The results showed a limit of detection of 10 mM for sweat, which falls within the range of lactate levels typically found in sweat. In another example, Joshi et al. [96] introduced a novel glucose/lactate FET sensing platform by immobilizing enzymes on a polyimide substrate [97]. In this approach, semiconductor material was made from carbon nanotubes, which randomly sprayed onto a Kapton membrane [98]. The drain current (IDS) for the p-type CNTFET increased as the concentration of lactate or glucose rised [99]. The devices exhibited good shelf life and the ability to withstand repetitive mechanical deformations, making it promising for non-invasive monitoring of biomarkers on wearable devices.

#### Antibody and nanobody (immunoFET)

**Fig. 9:**
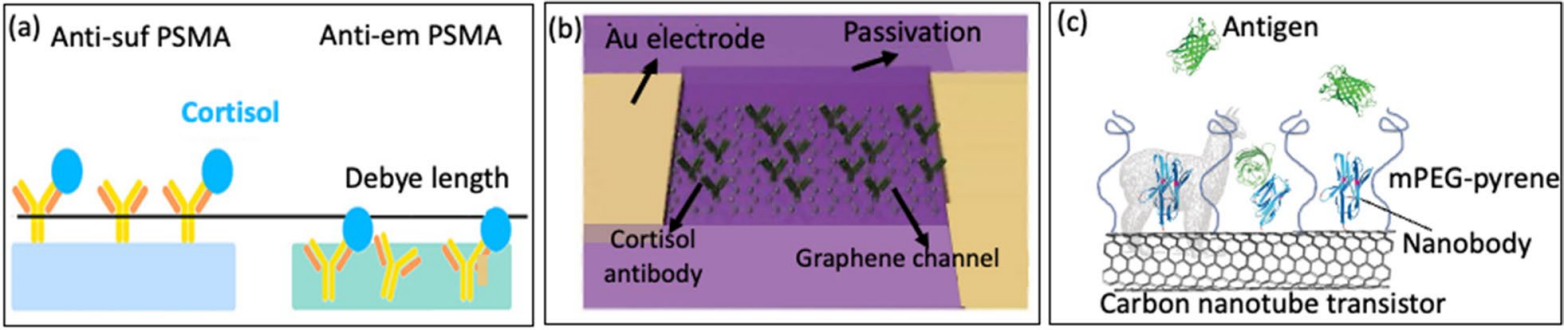
**a** Presumed schematic image of cortisol antibody-embedded geometric in the PSMA polymer matrix (reprinted with permission from Ref. [85] Copyright 2018 American Chemical Society). **b** Schematic image of the graphene FET device functionalized with cortisol antibody on the surface of graphene channel (reprinted with permission from Ref. [2] Copyright 2020 American Association for the Advancement of Science). **c** Representative carbon nanotube FET functionalized with nanobody (reprinted with permission from Ref [80] Copyright 2018 Elsevier)

Antibodies are essential protective proteins secreted by B-lymphocytes. They are characterized by their “Y” shape and play a crucial role in the immune response of mammals. Furthermore, Antibodies exhibit a remarkable ability to bind to specific antigens with high selectivity, making them valuable for detecting target-specific analytes/antigens [93–95]. The recognition between antibodies and antigens will generate an electric field as they are both charged molecules. This electric field alters the flow of carrier between the source and the gate, creating a signal that can be detected electrically [100, 101]. Therefore, the concentration of the analyte can be measured through changes in conductivity resulting from the interaction of antigen–antibody bonds [102], making it promising for clinical diagnosis applications. However, antibodies still have limitations due to its reach over Debye length (λ_D_), which can lead to decreased sensitivity. Jang et al. [85] made significant advancements in overcoming the issue of λ_D_ by fabricating FET- based sensor using antibody- embedded poly (styrene-co-methacrylic acid) (PSMA) sensing gate to detect cortisol, Fig. 9a. The embedded structure of the receptor in the polymer allowed cortisol molecules to bind near the membrane-substrate interface, effectively overcome the constraints posed by λ_D_. The authors compared its sensing efficacy with the traditional approach of antibody functionalization on the PSMA surface. With the aim of evaluating the performance of PSMA integrated with embedded antibodies. The antibody-embedded PSMA achieved a limit of detection (LOD) of 1 ng/mL in slightly buffered artificial sweat, demonstrating the potential to overcome the limitation imposed by λ_D_. The effectiveness of antibody-embedded PSMA was subsequently verified through a sandwich ELISA. This study is the first demonstration of FET-based cortisol sensing, where cortisol is electrically detected using polymers on a remote flexible gate platform. This creates opportunities for the detection of cortisol in saliva or sweat within a clinical setting.

Similarly, Chu et al. [103] developed a new type of FET biosensor using monoclonal antibodies (anti-CEA and anti-NT-pro BNP) to directly detect proteins beyond the discernable Debye length. Protein CEA was effectively detected by using antibodies immobilized on high-electron-mobility transistors (HEMTs). Initially, 2-Mercaptoethylamine (MEA) was utilized to break the disulfide bond in the heavy chain of the IgG antibody. The resulting cleaved thiol group then attached to the gold surface, aligning the antibody with a specific orientation. This coupling exposed the binding site upwards and reduced the distance between the binding and the gate electrode [84]. This approach managed to counteract the significant charge-screening effect caused by the high ionic strength in the solution. Because sensing is not dependent on charge of target proteins, it can detect both charged and neutral proteins. More importantly, this process does not require dilution or washing, making it simpler and more efficient. The practical approaches including evaluating designs, measurement methodologies, and the working mechanism of enzyme FET promise direct protein detection in diagnostics.

Ku et al.[2] fabricated GFET biosensors for cortisol detection by using monoclonal antibody (C-Mab) chemically bonded to the surface of graphene. Figure 9b depicts FET biosensors integrated with contact lenses to noninvasively monitor tears in real time. In their study, graphene serves as a transducer to turn the antibody-cortisol interaction into electrical signals. This approach was able to detect cortisol with an LOD of 10 pg/ml, which is within the typical range of human tear’s cortisol level from 1 to 40 ng/ml [104].

More recently, a novel approach to immunoFETs has emerged, where nanobodies are used to overcome the limitations of the Debye length (λ_D_). Nanobodies are stable and easily producible biological probes, characterized by their remarkably short length of less than 3 nm, which sets them apart from typical antibodies (15 nm) and even antibody fragments (7–8 nm). Their short length means these nanobodies can support analyte binding in proximities much closer to the surface of the sensor. Moreover, nanobodies exhibit impressive physicochemical stability under diverse conditions. As a result, researchers have integrated nanobody receptors into carbon nanotube transistors, giving rise to highly sensitive, selective, and label-free protein detection in physiological solutions [105]. Even though nanobodies possess distinct attributes, they have not been widely employed as probes in FET-based biosensors [106]. Among the few to use them, Filipiak et al. [80] proposed a novel surface modification approach to develop FET sensor by using a very short nanobody receptors combined with a polyethylene glycol layer to overcome the issue of the Debye length, Fig. 9c. Nanomaterial-based FETs with green fluorescent protein was used as the model antigen. FET sensors after being functionalized exhibited exceptional efficiency, sensitive, selective, and label-free protein detection across a wide range of concentrations. This capability remains consistent even in physiological solutions with a high ionic strength (100 mM).

#### Aptamer

**Fig. 10:**
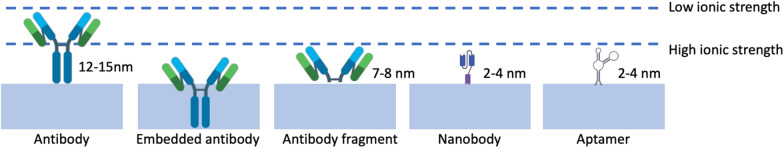
Several strategies to overcome the limitation of Debye length of immunoFET

Aptamers are short single-stranded oligo-nucleotide sequences of 15–40 nucleotides in length that have been engineered through a selection process to exhibit an exceptional binding affinity in a manner similar to antibodies and nanobodies [107]. One of the key advantages of aptamers is their small size, which is approximately one-tenth that of an antibody. This makes them potentially ideal for overcoming the Debye length limit when they interact with the target, enhancing sensitivity and lower detection limits, Fig. 10.

Aptamers offer several superior advantages over antibodies as catch probes. First, they are synthesized in vitro, reducing the variation between batches. Furthermore, aptamers can be designed to display varying degrees of affinity for a targeted molecule [108, 109]. Moreover, they demonstrate greater resilience to temperature fluctuations and remain stable during long-term storage [110, 111]. Furthermore, aptamers can be covalently immobilized on most surfaces by modifying either the 5′- or 3′-end [112].

Aptamers provide an additional advantage to apta-FET compared to immuno-FET. Unlike traditional bio-FETs that require target molecules to be charged, aptasensors can accommodate electroneutral targets through conformational changes in the aptamer's negatively charged phosphodiester backbones near the semiconductor channel surface. Target binding induces a secondary conformational shift, causing the aptamer to adopt a more compact structure that positions the negative charges closer to the semiconductor surface, resulting in a negative top gating effect on the device [113].

While the sensing mechanism of aptamer-FETs hinges on altering aptamer conformations due to target-induced surface charge redistribution (aptaswitch), gate voltage manipulation can also influence aptamer configurations [32]. This allows for the modulation of aptamer states to release targets and to achieve effective biosensor regeneration for continuous analyte monitoring. [114, 115].

**Fig. 11:**
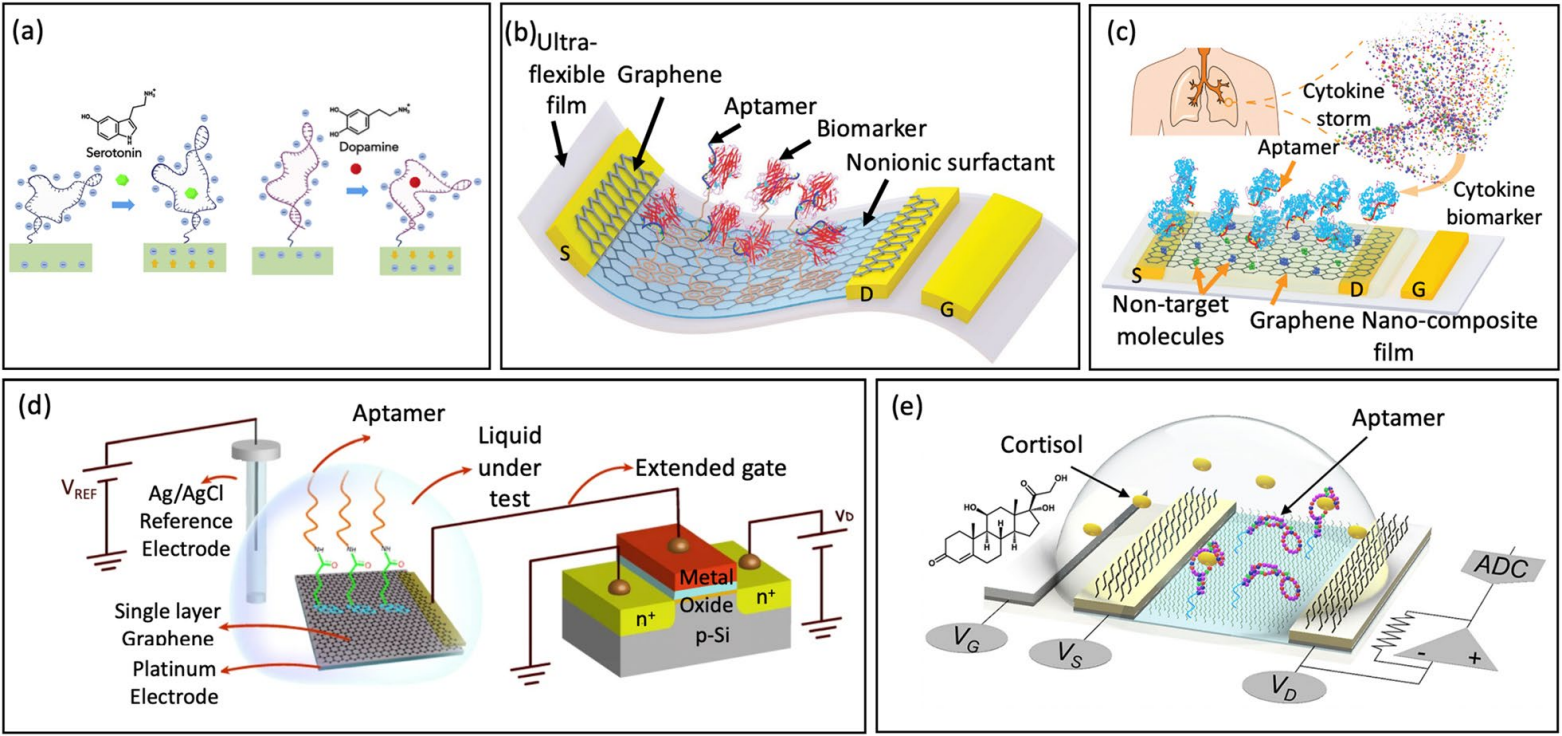
**a** Serotonin- and Dopamine-Aptamer-Functionalized FET sensors (reprinted with permission from Ref [33] Copyright 2020 Sciencedirect). **b** Schematic of the ultraflexible aptameric GFET nanosensor (reprinted with permission from Ref [16] Copyright 2019 Wiley). **c** Schematic of the aptameric GNFET biosensor for cytokine biomarker detection (reprinted with permission from Ref [118] Copyright 2020 Wiley). **d** Schematic of using a single layer of graphene as a gate electrode functionalized with aptamer (reprinted with permission from Ref [108] Copyright 2021 Nature). **e** Schematic illustration of cortisol sensing by an aptaFET sensor (reprinted with permission from Ref. [17] Copyright 2022 American Association for the Advancement of Science)

Liu et al. [33] introduced a platform of nanoribbon In_2_O_3_ FETs, which were functionalized with aptamers for monitoring serotonin and dopamine at varying concentrations. When the aptamer captures the target, a segment of the negatively charged backbone of the serotonin aptamer moves away from the In_2_O_3_ surface. Consequently, electrostatic repulsion between the electrons in an n-type semiconductor and the negatively charged aptamers would decrease, leading to an increase in channel conductance in response to association between the aptamer and the target. In contrast to the dopamine aptamer used in the study, the authors hypothesized that a section of the negatively charged backbone would move closer to the n-type semiconductor upon dopamine binding. This change increased electrostatic repulsion and decreased In_2_O_3_ transconductance, Fig. 11a. The device detected serotonin and dopamine over a broad concentration ranges, including those occurring in the brain extracellular space [116, 117], in real time, and in a multiplexed format that included temperature and pH sensing.

Wang et al. [16] employed a similar approach to fabricate an FET nanosensor by creating a conducting channel with monolayer graphene functionalize aptamer. The sensor was designed to detect TNF-α, an inflammatory cytokine biomarker, Fig. 11b. The interaction between the aptamer and the biomarker leads to a modification in the concentration of graphene carrier. An applied voltage between the drain and source terminals causes a current to flow through the graphene channel. This current is measured via the FET to determine the concentration of the biomarker. The nanosensor demonstrates consistent high selectivity and low LOD (down to 5 × 10^–12^ M TNF-α), making it potentially useful in wearable sensors.

Subsequently, Wang et al. [118] developed a cytokine sensing platform to assist hospitals in maximizing the benefits of anti-inflammatory therapies while avoiding cytokine storms, Fig. 11c. Existing sensors face challenges in accurately measuring cytokine levels in biofluids due to high background interference. To overcome this issue, the authors created an aptameric FET sensor using a composite graphene-Nafion film. This composite film minimizes nonspecific adsorption and increases renewability in the biosensor. With these advancements, the platform was capable of consistently and sensitively monitoring cytokines in undiluted human sweat, with a detection range from 0.015 to 250 nm and an impressive LOD down to 740 fM. Moreover, the device exhibited no visible mechanical damage and maintained a consistent sensing response during regenerative tests and crumpling tests. These advantages make it promising for extensive utilization in patients with acute infectious disease as well as conditions that require daily monitoring.

Furthermore, Sheibani et al. [108] made significant progress in wearable EG-FET sensor development to address the Debye screening limitation of charge sensing, using a single layer of graphene as a gate electrode and aptamers, Fig. 11d. Atomically thin graphene chemically binds with the aptamer and allows for the recognition event of the analytes occurring within the Debye length. Meanwhile, aptamers function as the recognition components, making the sensor remarkably sensitive, specific, and enduring. The EG-FET sensor is hysteresis-free and exhibits high selectivity towards other similar hormones with a detection limit of 0.2 nM.

In fact, the system integration of FET-based biosensors has not been significantly developed and restrict their adaptation for wearable applications [85, 119, 120]. Wang et al. [17] fabricated a fully integrated sensing platform comprising novel cortisol aptamer binding with a nanometer-thin-film. The cortisol aptamer, which has a thiol modification at the 5′ end, was covalently binded to amino-silanized In_2_O_3_ FET. The cortisol aptamer, which had a thiol modification at the 5′ end, was covalently binded to amino-silanized In_2_O_3_ FET, Fig. 11e. The sensing system operates autonomously and wirelessly, label-free and remarkably low cortisol detection limits highlight the potential of monitoring sweat cortisol for practical applications [121, 122]. They can be transformed into wearable and mobile formats to cater to other physiological biomarkers. This is especially valuable for targets present with low concentrations in sweat, where portable measurement technologies are currently lacking. This can contribute to the progression of personalized precision medicine.

#### Ion-sensitive membrane

Ion-selective membranes are a highly effective method of chemical sensing. The membrane has specific ion receptors (ionophores). The membrane is placed on top of the gate stack of the sensing dielectric. The ionophore in the membrane selectively interacts with its target ion, while showing weaker interactions with interfering ions. A junction voltage is developed as a result of these interactions. This voltage not only affects the gate bias of the ion-sensitive FET but is directly proportional to the ion activity at the liquid-to-ISM interface [1].

To achieve selective sensitivity to different ions, the sensing dielectric is functionalized with an ion-selective membrane for each ion species. This means that only the specific ion for which the membrane has been functionalized can pass through and provide its charge to the ion-sensitive FET's gate [1]. In this way, ion-selective membranes allow different ions to be detected precisely and selectively for chemical sensing applications.

Ion-sensitive FETs (ISFETs) hold great promise for continuous monitoring of biofluids in real time. However, the development of wearable sensors involving ISFETs has been hindered by the need for bulky reference electrodes. A stable reference electrode is essential for ion detection since it must maintain a constant potential under varying ion concentrations to ensure that the sensors function properly.

**Fig. 12:**
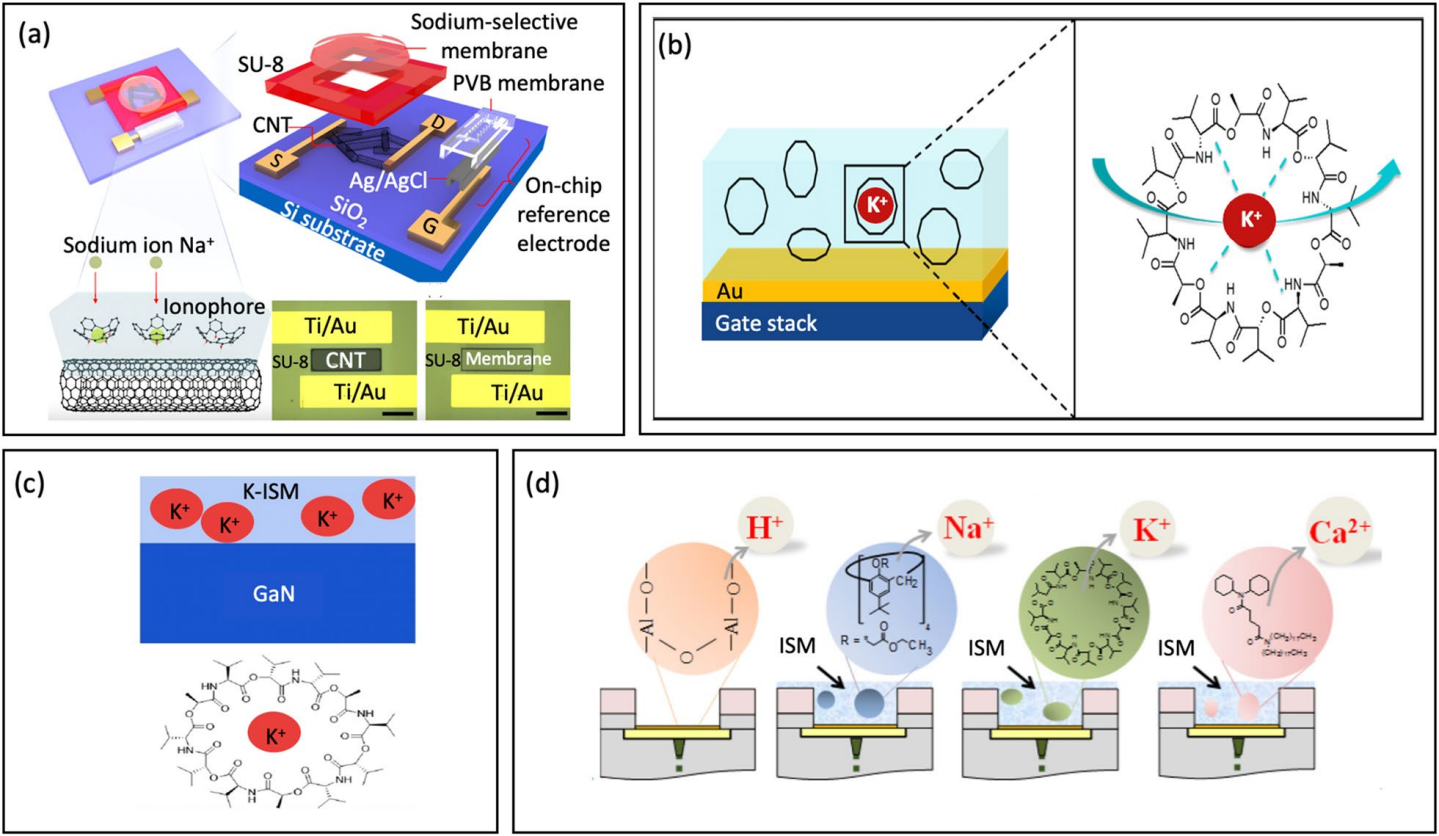
**a** Schematic of CNT-FETs with the sodium-selective membrane and on-chip reference electrode (reprinted with permission from Ref. [123] Copyright 2021 American Chemical Society). **b** Functionalization chemistry of Au gates with a polymeric membrane for potassium sensing (reprinted with permission from Ref. [19] Copyright 2018 American Chemical Society). **c** Potassium ion-selective membrane modified on the GaN surface (reprinted with permission from Ref. [127] Copyright 2019 Wiley). **d** 3D-EMG-ISFETs with Al_2_O_3_ as pH sensing dielectric, functionalized with ion selective membranes for Na^+^,K^+^, and Ca^2+^ sensing (reprinted with permission from Ref. [1] Copyright 2019 American Chemical Society)

To address this limitation, Park et al. [123] introduced a novel carbon nanotube FETs (CNT-FETs) platform using ion-selective membrane and a miniaturized on-chip reference electrode for sodium sensing, Fig. 12a. The CNT surface is modified with a sodium-selective membrane made of polyvinyl chloride with a specific ionophore, which selectively captures sodium ions with high sensitivity. The electrochemical potential generated in the membrane is then converted into a channel current for the CNTs. A miniaturized reference electrode was integrated to achieve a compact size for wearable devices, which was not previously accomplished by commercial reference electrodes. The on-chip reference electrode has a stable performance, outperforming conventional reference electrodes. The sodium sensor was capable of selectively detecting sodium ions over a wide concentration range from 0.1 to 100 mM. This range covers typical human sweat sodium concentrations, even in the presence of interfering ions such as magnesium, calcium, and potassium.

ISFET devices have emerged as a promising alternative to other sensing technologies due to their ability to integrate with a range of electronic readouts. For this reason, these devices are considered to be highly versatile and multifunctional. For instance, Erick et al. [19] introduced a fully-integrated on-chip sweat sensing system that is both wearable and capable of tracking sodium and potassium levels, Fig. 12b. These are essential markers for identifying hormonal changes associated with ovulation [124] and cystic fibrosis [125]. The ratio between potassium and sodium concentration in sweat can also be indicative of kidney failure [126]. In this device, valinomycin was mixed with a PVC membrane to create a potassium ion-selective membrane, while Na ionophore X was mixed with a PCV membrane to create a sodium ion-selective membrane. The concentrations of sodium and potassium exhibited nearly Nernstian sensitivity, indicating that the functional membranes possess high sensitivity. Real-time measurements also indicated stable and repeatable readings. Their response times align well with the physiological rate of ion concentration variations in sweat.

Besides, the long-term stable and repeatable detection of ions has been a challenge for FET biosensors – one that has limited their adoption as a truly efficacious health-monitoring platform. To address this issue, Liu et al. [127] introduced a wearable platform based on AlGaN/GaN high-electron-mobility transistors (HEMTs) for continuously monitoring pH and potassium ions, Fig. 12c. The platform incorporates a sweatband that continuously collects sweat. Detecting units for pH and potassium ion are created by modifying the surface with different sensitive films. The GaN surface is embedded with K^+^ in the PVC film to form a potassium ion-selective membrane. The platform exhibited high sensitivity (45.72 μA/pH for pH 3–7, 51.073 μA/pH for pH 7.4–9, and 4.94 μA/lgα_K_^+^ for K^+^), solid stability (maintained over 28 days), and good repeatability (with a relative standard deviation (RSD) of 2.6% for pH 3–7 sensitivity, an RSD of 2.1% for pH 7.4–9 sensitivity, and an RSD of 7.3% for K^+^  sensitivity).

Notably, Zhang et al. [1] firstly presented ﻿multianalyte sensing platform ISFET capable of detecting four distinct analytes in sweat: pH, Na^+^, K^+^, and Ca^2+^ by integrating readout interface, NFC communications, and several remotely powered 3D extended metal gate ISFETs (3D-EMG-ISFETs), Fig. 12d. The entire platform occupies less than 2.5 mm^2^. Furthermore, highly selective ion-selective membranes coupled with postprocessing integration steps eliminate significant sensor hysteresis and parasitic, resulting in high sensitivity of 58 mV/pH, -57 mV/dec (Na^+^), -48 mV/dec (K^+^), and -26 mV/dec (Ca^2+^). This is close to the Nernstian limit and high selectivity. The platform demonstrated in-vitro usability by measuring multiple analytes simultaneously. Remarkably, the sensors boasted the lowest power consumption ever reported at 2 pW/sensor. This ultralow power consumption means the sensor, the readout interface, and the ISFET sensors can all be remotely powered by a radio frequency signal. As such, this platform demonstrates a great potential for predictive analytics and personalized medical treatment, making it a promising candidate for wearable health monitoring applications.

### Biomarkers in physiological fluids

Table 3 presents list of potential biomarkers including tears, saliva, sweat, ISF, and associated parameters.

**Table 3:** Various biomarkers detection using non-invasive wearable FET biosensors

Biofluids	Biomarker	Disease/application	Clinical range	Sensing probe	Gating	Semiconductor	Dynamic range	Refs.
Sweat	Ammonia	Muscular fatigue	0.5–25 mM [128]	Ion-selective membrane	Top-gate	SWCNT	0.01–10 mM	[41]
	Na + /K + /Ca2 +	Monitoring sweat electrolytes	Na^+^:10-140 mM [129] K^+^:2-6 mM [129] Ca^2+^:30-300 mg/L [130]	Ion-selective membranes	3D extended metal gate	SiNW	Na^+^:1-100 mM K^+^:1-50 mM Ca^2+^: 0.1-20 mM	[1]
	Na + /K +	Ovulation, cystic fibrosis, kidney failure	Na^+^: 10-140 mM [129] K^+^: 2-6 mM [129]	Ion-selective membranes	Top-gate	MOS	Na^+^: 5-100 mM K^+^: 5-50 mM	[19]
	Na +	Monitoring sweat electrolytes	10-140 mM [129]	Ion-selective membranes	Side-gate	SWCNT	0.1-100 mM	[123]
	Lactate	Monitoring lactate level	2–65 mM [131]	Lactate oxidase, horseradish peroxidase and Osmium-redox polymer	Extended-gate	pBTTT-C16 (organic semiconductor)	LOD: 66 nM; LOQ: 220 nM	[132]
	s-IgA	Depression	1230 pg/mL [133]	Jacalin	Liquid-gate	Si	0.1–100 μg/mL	[77]
	Cortisol	Stress-related disorders	0.1–139.9 ng mL[133]	Aptamer	Side-gate	In_2_O_3_	1 pM-10 μM	[17]
	TNF-α	Inflammatory cytokine	9.3–21.1 pg/ml [134]	Aptamer	Side-gate	Graphene	50 pM-100 nM; LOD: 5 pM	[16]
	Cortisol	Stress-related disorders & human performance	0.1–139.9 ng/mL [133]	Aptamer	Extended-gate	Graphene	﻿1 nM—10 µM	[108]
	Serotonin and dopamine	Monitoring multiple neurotransmitters	Not given	Aptamer	Side-gate	In_2_O_3_	10fM-1uM	[33]
Tear	Glucose	Diabetes	0.1–0.6 mM [135]	Glucose oxidase	Back-gate and liquid gate	Graphene	1 µM to 10 mM	[93]
	Glucose	Diabetes	0.1–0.6 mM [135]	Glucose oxidase	Back-gate	MoS_2_	0.1–0.6 mM	[32]
	Cortisol	Stress-related disorders & human performance	0–1306 nM [133]	Antibody	Liquid-gate	Graphene	1–40 ng/mL; LOD: 10 pg/mL	[2]
	TNF-α and IFN-Y	Monitoring inflammatory cytokines	TNF-α: 1.1–21.7 pg/mL [136] IFN-Y: 3–12 pg/mL[137]	Aptamer	Side-gate	Graphene	TNF-α: 0.03–500 nM, LOD: 2.75 pM INF-Y: 0.03–500 nM, LOD: 2.89 pM	[138]
	Matrix metalloproteinase-9	Chronic ocular surface inflammation	3–41 ng/mL [139]	Antibody	Back-gate and liquid gate	Graphene	1–500 ng/ml	[140]
Saliva	Ammonia	Metabolism, mouth bacteria	Not given	Ion-selective membranes	Liquid-gate	zinc titanium oxide	10^–4^ – 1 M	﻿[21]
	Cortisol	Stress-related disorders and human performance	0.1–139.9 ng/mL [133]	Aptamer	Liquid-gate	Graphene	0.01–10^4^ nM	[81]
	Oxytocin	Monitoring of neurotransmitter; lactating women	6.44–61.05 pg/ml [141]	Antibody	Extended-gate	C6-DNT-VW/polystyren (organic semiconductor)	LOD: 3.9 pg/mL	[40]
	Carbonic anhydrase 1	Cancers, pancreatitis, diabetes and Sjogren's syndrome	Not given	Aptamer	Liquid gate	Graphene	10 pg/ml-100 ng/ml	[142]
ISF	Na^+^	Dysnatremia	145 mM [143]	Ion-selective membranes	Extended-gate	not given	10–160 mM; LOD: 2.78 μM	[144]
	C-Reactive Protein	Inflammation	Not given	Antibody Fab fragment	Back-gate and liquid gate	SiNW	60 ng/mL-100 μg/mL	[35]
	Glucose	diabetes	2.1–23.4 mM [145]	Glucose oxidase	Back-gate	ZnO	1 μM-10 mM	[44]

#### Sweat-based sensors

Sweat contains a variety of biochemical compounds, including ions [146], metabolites [147], acids [148, 149], hormones [150, 151], small proteins [152, 153] and peptides, along with a rich distribution of sweat glands (> 100 glands/cm2). Recent studies demonstrated that biomarkers in sweat are directly correlated with their concentrations in blood, rendering sweat a promising biological fluid for non-invasive diagnostics [1, 9, 154].

For instance, Petrelli et al. [41] developed an ammonium sensing platform using an electrolyte-gated carbon nanotube FET (EG-CNTFET). This platform provides a real-time profile of ammonium sweat dynamics, which are being explored as potential markers for the onset of muscular fatigue [155]. While most studies on ammonium sensing in sweat have used electrochemical sensors [156], the authors opted for a different approach using EG-CNTFETs functionalized with an ion-selective membrane instead. This is due to the unique properties of single-walled carbon nanotubes (SWCNTs), including high surface-to-volume ratio, chemical stability, and the ability for solution-processing. Additionally, EG-FET platform is well-suited for detecting analytes in a liquid phase [157]. The sensors yield a linear characteristic of ammonium in the range from 0.01 to 10 mM.

**Fig. 13:**
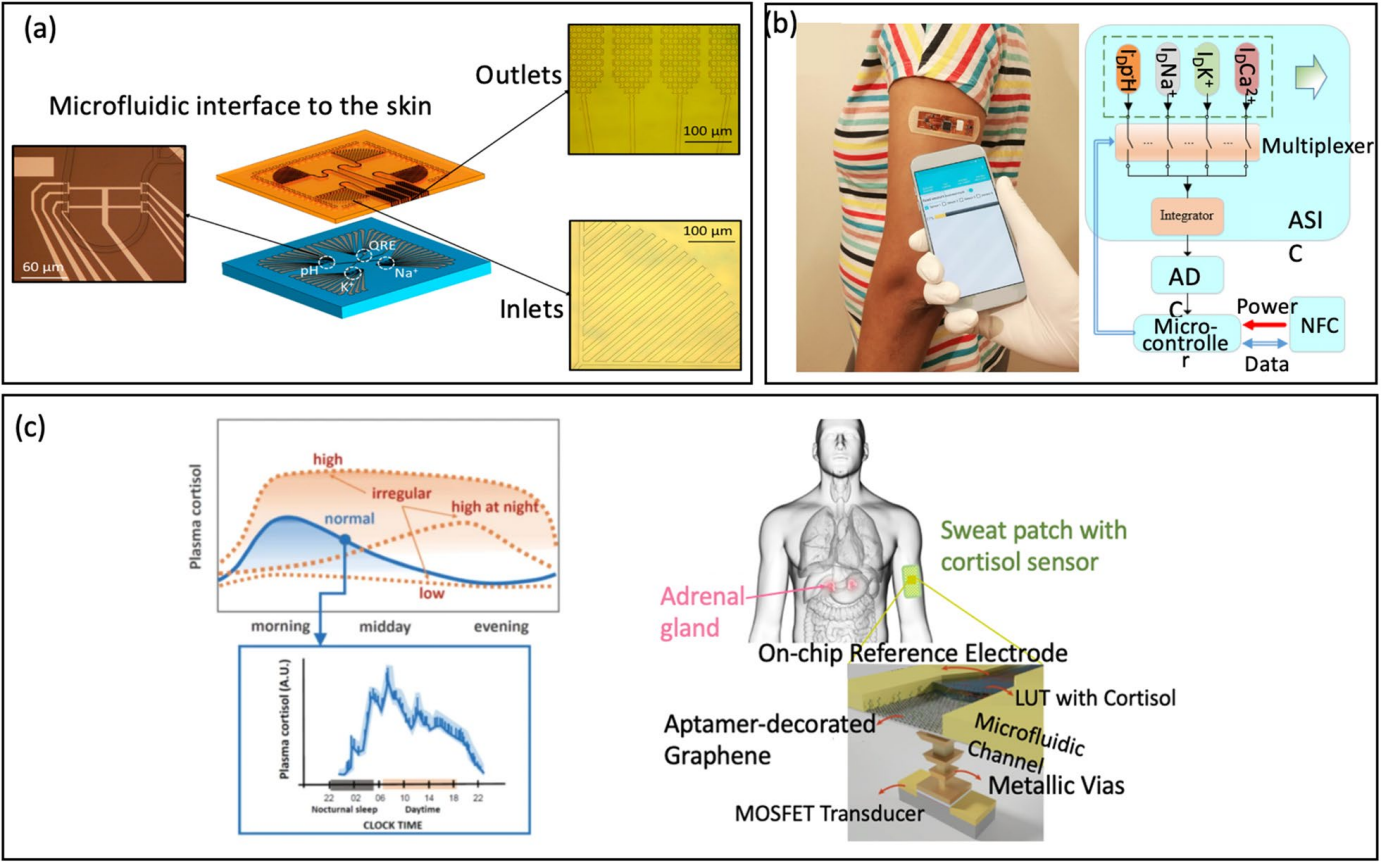
**a** Representative of lab-on-skin concept (reprinted with permission from Ref. [19] Copyright 2018 American Chemical Society). **b** Photo of the wearable system and Block diagram of the NFC powered sensing system (reprinted with permission from Ref. [1] Copyright 2019 American Chemical Society). **c** Qualitative depiction of regular and irregular circadian levels of the cortisol produced by the adrenal glands in human body through the day, showing the need for high time granularity measurements to capture the pulsatile nature of cortisol, and concept of 3D-integrated cortisol sensor integrated with a top microfluidic channel that guides the sweat over a planar reference electrode and bio-functionalized graphene (reprinted with permission from Ref [108] Copyright 2021 Nature)

In another study, Garcia-Cordero et al. [19] demonstrated ion-sensitive FETs sensor to monitor biochemical information in real-time from the skin surface, Fig. 13a. This system collects a small amount of sweat from a person's skin. Subsequently, the collected sweat was passively transported to a group of functionalized ion-sensitive FETs (ISFETs) where it was analyzed for its pH levels and the concentrations of Na^+^ and K^+^ ions. The combination of a microfluidic interface with an ISFET make it more convenience to collect even in low-sweat-rate conditions, or very small amounts of sweat. In this manner, the device can analyze sweat even during periods of rest, rendering it an exceptionally practical and convenient method for continuously monitoring biochemical markers.

Moreover, Hayashi et al. [77] developed wearable FET sensors for non-invasive detection of immunoglobulin A (s-IgA) in human sweat, a biomarker for certain mental health conditions, particularly depression. In fact, measuring through sweat face limit due to non-specific binding of substances such as sebum, mucin, proteins, and bacteria, leading to a reduction in the specificity of the sensor [152, 158, 159]. To overcome this challenge, the team immobilized jacalin as a receptor, which specifically allows for s-IgA to be adsorbed through filtration process, Fig. 13b. These jacalin-immobilized FET biosensors demonstrated higher sensitivity, operating in a range from 0.1 μg/mL to 100 μg/mL, making it a promising candidate for monitoring stress and analyzing s-IgA levels in a non-invasive manner. Recently, Sheibani et al. [108] developed a label-free FET sensor for detecting cortisol in a Debye screening time frame within a biological buffer, Fig. 13c. The wearable FET sensor employs an extended-gate aptamer based on platinum/graphene (EG-FET) that is hysteresis-free and possesses high voltage and current sensitivity. The results indicated a detection limit within the range of cortisol concentrations in fluids, rendering it suitable for continuous real-time monitoring of cortisol in human sweat.

#### Tear-based sensors

Tears are complex extracellular fluids comprising proteins, peptides, electrolytes, lipids, and metabolites that originate from a variety of sources such as the lacrimal glands, epithelial cells on the surface of the eye, Meibssomian glands, goblet cells, and blood [9]. As tears contain several components like those in blood, it’s possible to use tears to monitor biomarkers. However, the extraction and analysis of tears in vitro pose several challenges, which have prevented tears from being used as a diagnostic tool. First, tear sample can be evaporated during transport to a laboratory, significantly impacting the accuracy of centralized tear analyses. Second, the human eye is delicate, so great caution is needed when collecting samples. Additionally, biomarker concentrations often vary depending on the specific collection method in use, which may undermine any test findings [160, 161]. Thus, smart contact lenses have garnered significant interest, and the integration of biosensors with contact lenses is a promising approach for real-time monitoring and assessment of health condition [93, 162–164].

**Fig. 14:**
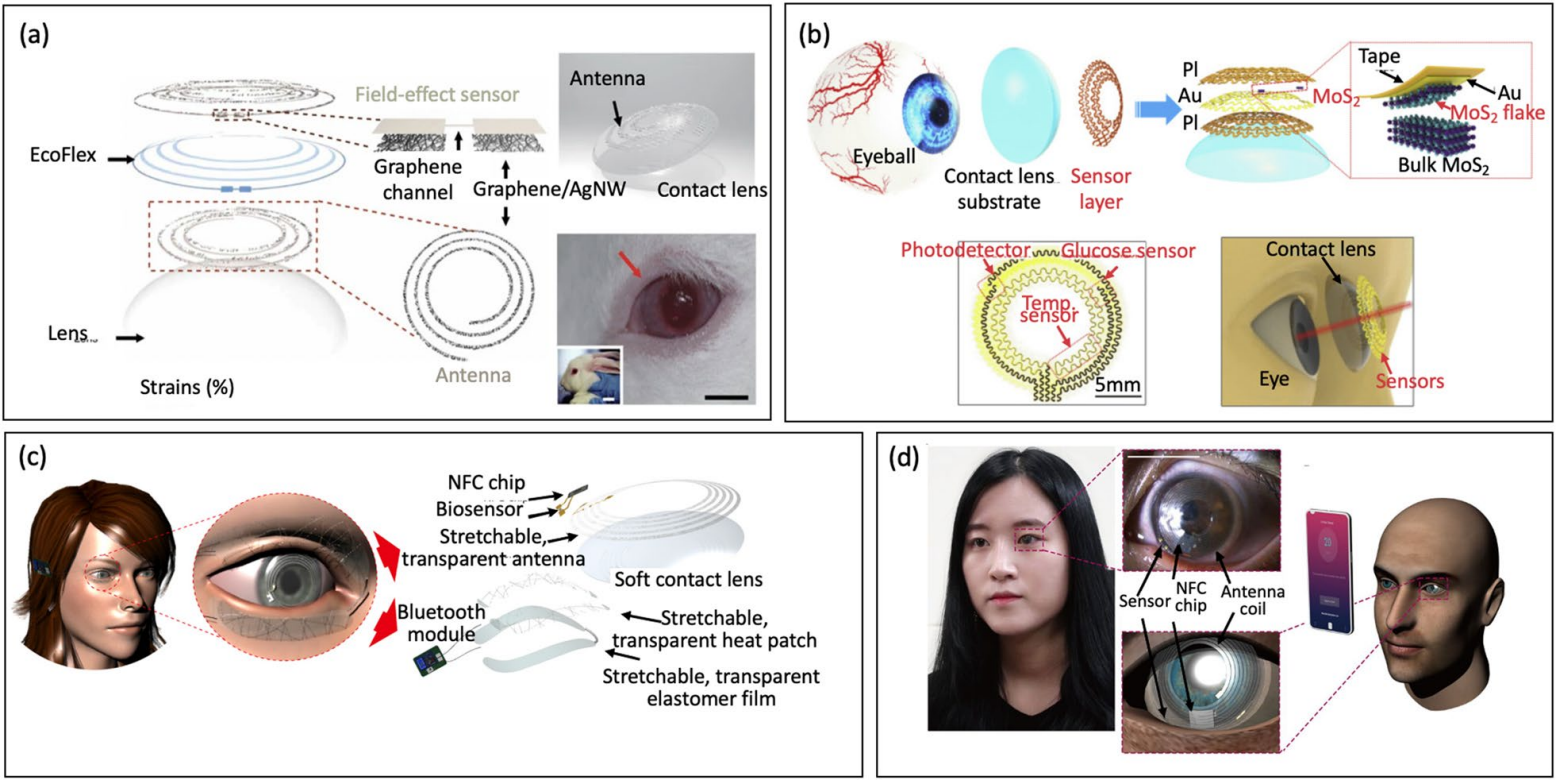
**a** Schematic of the wearable contact lens sensor, integrating the glucose sensor and intra-ocular pressure sensor (reproduced with permission from Ref. [93] Copyright 2017 Nature). **b** Structural design of a smart contact lens with ultrathin MoS_2_ transistor-based serpentine mesh sensor (reprinted with permission from Ref. [32] Copyright 2021 Elsevier). **c** Schematic illustration of the integrated system of the diagnostic and therapeutic devices for the real-time monitoring and therapy of chronic operational stress injury (reprinted with permission from Ref. [140] Copyright 2021 Science). **d** Photograph of an adult wearing the smart contact lens on her eye for cortisol level monitoring (reprinted with permission from Ref. [2] Copyright 2020 Science)

However, contact lens sensors face some serious limitations such as obstructing user’s vision and lacking capability of multiplex analysis. To overcome some of these shortcomings, Kim et al. [93] integrated a sensor into an actual ocular contact lens, Fig. 14a. The sensor comprises of a transparent graphene hybrid with metal nanowires, which offers transparency and stretchability to ensure user comfort without obstructing their line of sight to detect glucose in tears continuously and wirelessly. As a multifunctional device, the lens can also measure intraocular pressure, which is linked to glaucoma. In-vivo and in-vitro tests on rabbits and bovine eyeballs demonstrate consistent and dependable performance. Although this device allows for multifunctional sensing, the authors have not yet demonstrated both functions working concurrently. Likewise, Guo et al. [32] developed a multifunctional contact lens equipped with a flexible MoS_2_ FET mesh sensor to detect glucose, temperature, and UV light, Fig. 14b. Unlike traditional sensors embedded within lens substrates, the authors directly affixed a serpentine mesh sensor system onto the lenses to maintain direct contact with tear fluid. This approach allows for high detection sensitivity, while ensuring mechanical robustness and no interference with blinking or vision. The results showed good photo-detection response, high-sensitivity glucose detection, and accurate temperature measurement, making it a promising candidate in healthcare applications.

While the majority of smart contact lenses mainly focus on monitoring glucose levels or intraocular pressure, Jang et al. [140] developed a graphene FET biosensor to quantitatively diagnose ocular surface inflammation (OSI) based on the concentration of MMP-9 in tears, Fig. 14c. By integrating a smart contact lens with a skin-attachable therapeutic device, the system can wirelessly monitor through tears. The FET biosensor is integrated with a wireless antenna, capacitors, resistors, and an NFC chip via stretchable interconnects to ensure that it does not hinder the wearer's field of vision. The results are wirelessly transmitted to the user's mobile device in real-time, allowing non-invasive diagnosis of OSI. Furthermore, Ku et al. [2] developed a smart contact lens to detect cortisol in tear. The graphene FET sensor was fabricated by immobilizing a monoclonal antibody (C-Mab) onto the graphene surface, Fig. 14d. The platform was able to measure cortisol concentrations in real time with a detection limit of 10 pg/ml, within the range in human tears. Integration of cortisol sensor with a transparent antenna and wireless communication circuitry enables smartphones remotely controlled without obstructing the user's line of sight. In vivo tests using live rabbits and human subjects confirmed the excellent biocompatibility and reliability of the lens.

#### Saliva-based sensors

**Fig. 15:**
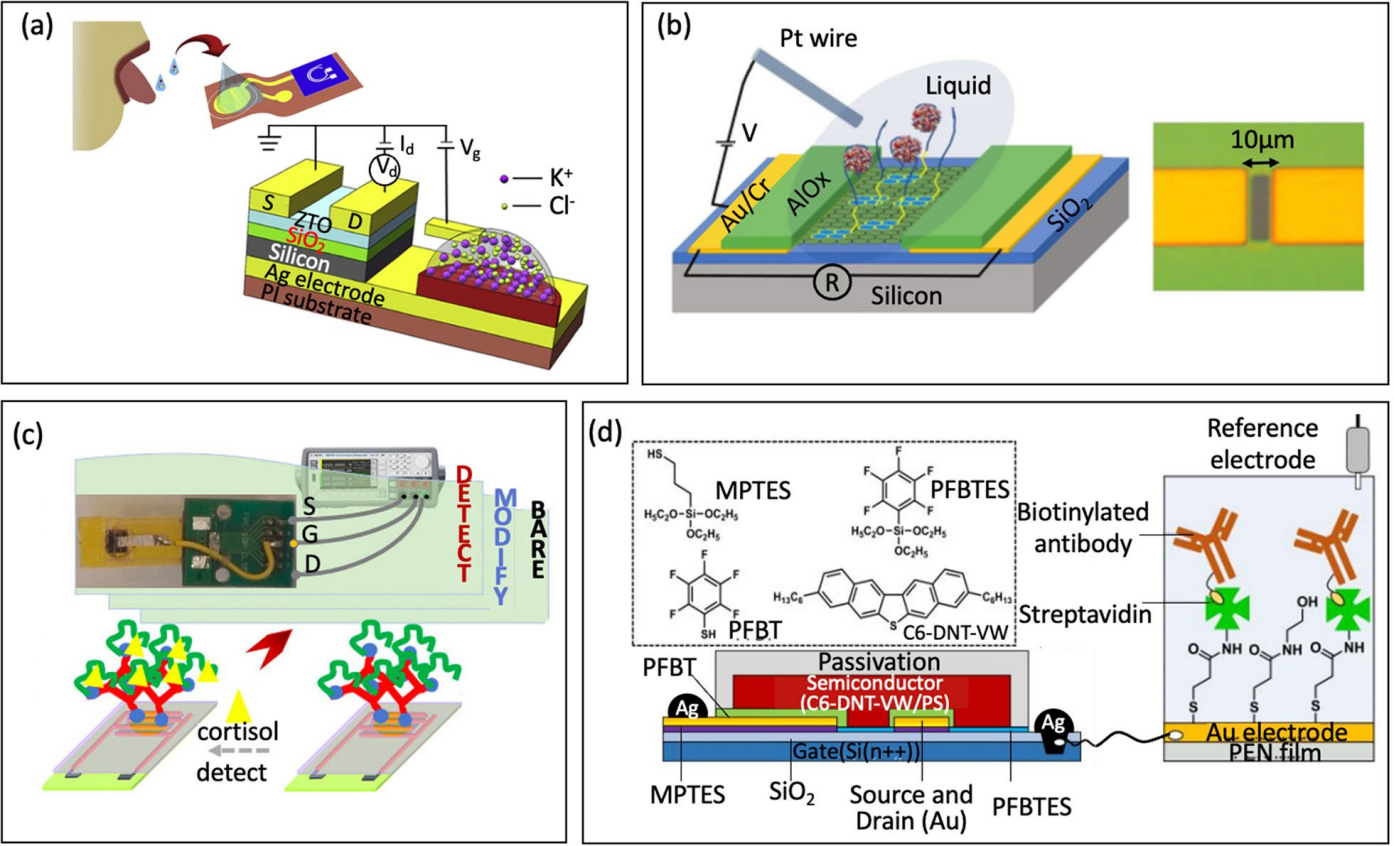
**a** Schematic of the application for selective ion detection in artificial saliva ISFET (reprinted with permission from Ref. [21] Copyright 2019 Elsevier). **b** Schematic representation of a G-FET device utilized for biosensing (reprinted with permission from Ref. [142] Copyright 2020 Willey). **c** The device fabrication processes and device tests for evaluating the electronic features of the bare Lg-GFETs (reprinted with permission from Ref. [81] Copyright 2021 American Chemical Society). **d** Schematic illustration of the extended-gate-type OFET sensor for oxytocin detection (reprinted with permission from Ref. [40] Copyright 2022 RSC)

Saliva is an oral fluid primarily produced by the parotid gland. Saliva comprises various components, such as metabolites, hormones, enzymes, microorganisms, proteins, and ions [165, 166]. Saliva is commonly sampled through passive drooling directly into a device [167] or with the use of a swab [168]. Integrating saliva sensors with devices placed in the mouth, such as toothbrushes [169], mouthguards [170], pacifiers [171], and even teeth, allows for in-situ sampling and detection of analytes. In-mouth biosensing platforms offer a painless and convenient method for obtaining real-time chemical information from saliva. Despite its potential for monitoring health, not many developments of wearable oral biosensors were reported in literature. In their pioneering work, Bao et al. [21] introduced an integrated wearable healthcare platform for monitoring ions, such as ammonium (NH_4_^+^), potassium (K^+^), and calcium (Ca^2+^). The platform was fabricated using 3D printing methods, which allows for a direct connection between the oxide FETs and ion-sensitive electrodes, resulting in the formation of hybrid ISFETs, Fig. 15a. The test results on artificial saliva showed that reported ISFET exhibits high sensitivity and selectivity even in interfering ions environments.

Similarly, Kumar et al. [142] developed oral biosensing to detect human carbonic anhydrase 1 (CA1)—a biomarker for diagnosing several diseases such as diabetes, pancreatitis, cancers, and Sjogren's syndrome, Fig. 15b. Graphene-based FETs functionalized with RNA aptamer were employed with liquid gating to minimize voltage range, preserving original structures and aptamers. The G-FET biosensors exhibited a low detection limit of 10 pg/ml in just 30 min. Likewise, Zhang et al. [81] introduced a portable salivary cortisol test using liquid gate graphene FET (Lg-GFET), as shown in Fig. 15c. This sensor not only has excellent dynamic range of seven logs (0.01 to 10^4^ nM), but it also has strong anti-interference capabilities to distinguish between substances with similar chemical structures. The combination of the cortisol sensor platform, measuring block, and installed application on mobile phones allows users to conveniently use it at home. In addition, Ohshiro et al. [40] developed extended-gate organic FET sensor detect oxytocin in saliva, Fig. 15d. Through a functionalized extended-gate electrode, the device can discriminate between hormones with only slightly different chemical structures, achieving accurate oxytocin detection with a LOD value of 0.57 pg/mL.

#### Skin interstitial fluid-based sensors

Skin interstitial fluid (ISF) is the fluid surrounding cells, through the process of diffusion, ISF maintains continuous equilibrium with the blood capillaries, serving as a bridge between blood and cells [172]. ISF is the most accessible bodily fluid, primarily present in the subcutaneous tissue layer, constituting 70% of the volume [173]. ISF has been demonstrated to correlate with blood, containing significant information for monitoring physiological indicators and can be collected non-invasively and continuously [174]. The most detected biomarker remains glucose for diabetes diagnosis over past few decades.

Typically, measuring the concentration of substances in ISF is carried out with microneedles. These microneedles have proven to be highly efficient in measuring sodium levels in ISFs, without causing discomfort to the individual [175–177]. However, conventional microneedles often possess rigid structures, making them unsuitable for applications in devices that require elasticity or flexibility. To address this limitation, Zheng et al. [144] developed an extended-gate FET biosensor capable of stretching to detect sodium, a biomarker to minimal invasively diagnosis of dysnatremia. The FET sensor included an extended gate made of microneedles penetrating the skin to access ISF where sodium is measured. The reported device exhibited high sensitivity, real-time monitoring, excellent biocompatibility, low detection limits, and mechanical stability on the body. Recently, Capua et al. [35] developed an FET biosensor using silicon nanowire arrays to detect C-reactive protein in ISF. The authors used SiNW arrays immobilized with antibody fragments to overcome Debye screening and enabled label-free detection. Reference subtraction method was applied to ensure specific protein detection. The reported FET sensors facilitated real-time diagnosis and detected CRP within the range of physiological concentration 60 ng/mL to 100 μg/mL.

### Challenges and opportunity

#### Technical problems

Some FETs made of traditional materials are facing problems such as leaking currents, when a channel’s surface is not smooth enough at the nanoscale [178–181]. To address these challenge, Chhowalla et al. [182] listed three key features of FETs: (1) an ideal insulator material to prevent leakage current between the gate, source, and drain electrodes, (2) no leakage voltage drop between the contacts, and (3) a device design that avoids electron scattering in the channel. Additionally, environmental stability, controlled and stable doping, and uniform growth are essential for developing an effective FET.

Standardization also plays a vital role in the fabrication, characterization, and performance evaluation of FET biosensors. Standardization ensures the quality, reliability, and interoperability of these sensors. Currently, the lack of standardization poses challenges for comparing and reproducing FET sensors across different studies and platforms. To address this issue, establishing standard protocols and guidelines is essential. This can be achieved through reference materials, developing common metrics for evaluation, and sharing data and best practices among researchers [183]. By implementing standardization, researchers and developers can improve the consistency, comparability, and reliability of FET biosensors to advance practical applications in a range of fields.

Another challenge with epidermal electronics is that they typically involve complicated data processing circuits and need to communicate wirelessly, which are both difficult to implement [183]. One promising solution on the horizon are microwave sensors, which are based on changes in the electromagnetic properties of the sensing materials at ultrahigh frequencies (ca. > 1 GHz). Furthermore, novel bioenergy solutions are essential to transition from conventional laboratory-based sensing to efficient wearable biosensing. These may include self-powered wearable biosensing systems integrated with biofuel cells or battery-free options utilizing NFC technology [13].

#### Biological challenges

There are several challenges associated with analyzing biofluid with a small volume, which tends to evaporate quickly. Furthermore, there is a great risk of contamination, particularly when saliva is involved and may be mixed with food or drink residues. Additionally, the variability in collection time poses a challenge [9]. To address these issues, microfluidic systems have emerged, which not only offer precise control over sample manipulation but also reducing evaporation. Furthermore, incorporating permselective protective sensor coatings can help prevent the presence of macromolecules on the sensor surface.

Tear, saliva, sweat, interstitial fluids typically have lower concentrations of analytes than in blood samples, thereby requiring ultrasensitive biosensors to ensure accurate detection. It is also crucial to consider the biocompatibility of materials incorporated in these biosensors, particularly when dealing with tear and saliva samples, to ensure their safety for use in the human body. Finally, it may be necessary to undertake a comprehensive study involving a large number of clinical samples to validate the results in non-traditional fluids [184].

#### Challenges in system integration and hardware

The development and implementation of wearable FET biosensor devices involves integrating various components, including sensors, signal processors, data transmitters, and some form of power management, with the ultimate goal of enabling non-invasive, continuous, and accurate monitoring of biochemical markers in biofluids. Several approaches have been taken to build reliable, comfortable, and user-friendly wearable biosensor systems, such as flexible printed circuit boards, stretchable interconnects, wireless communication modules, and energy harvesting or storage devices [24]. However, there are several challenges and trade-offs that need to be addressed to achieve the desired reliability, comfort, and user-friendliness of wearable biosensor systems. First, a balance is needed between the size, weight, power consumption, and performance of the device components. This involves optimizing the design and selecting the components to ensure an optimal balance between these factors. Second, ensuring the biocompatibility and durability of the materials and sensors used in wearable biosensor systems is crucial. The materials should be compatible with the human body to prevent any adverse reactions, while also being durable enough to withstand everyday use and potential environmental factors. Third, maintaining the stability and accuracy of biosensing signals in different environmental and physiological conditions is a challenge. Wearable biosensor systems should be able to provide reliable measurements regardless of variations in temperature, humidity, motion, and other factors that may affect the signals. Lastly, ensuring the security and privacy of the data transmission and processing is of the utmost importance. Wearable biosensor systems should incorporate robust encryption and authentication measures to safeguard the transmitted data and protect the user's privacy [184].

#### Challenges in device stretchability

When it comes to developing wearable biosensors, a key aspect to consider is the mechanical mismatch between rigid active materials and soft human tissues/skin. Lyu et al. [12, 13] have provided a comprehensive review on soft wearable devices, highlighting two main strategies for achieving stretchable devices: deformable architectures and intrinsically stretchable materials. Deformable architectures involve designing structures that can deform or buckle under strain. Some examples of deformable architectures include buckling, microbelts, serpentine, holey, nanomesh, and kirigami. These designs allow the device to stretch and conform to the shape of the human body. On the other hand, intrinsically stretchable materials are materials that possess inherent stretchability. Commonly used stretchable materials for wearable devices include PDMS (polydimethylsiloxane), eco-flex, polyurethane, polyethylene terephthalate (PET), and polyimide. Leveraging deformable architectures and intrinsically stretchable materials, researchers and engineers can develop wearable biosensors that are flexible, comfortable to wear, and capable of accurately monitoring various physiological parameters.

#### Commercialization challenges and opportunities

Commercial sensors are expected to meet a range of demands, including sensitivity, reliability, scalability, affordability, data security, and real-time communication capability [24]. These factors are essential for the successful adoption and commercialization of sensors across various industries. In addition, future prospects of wearable FET biosensor devices in terms of commercialization depends on the existing products and companies involved in their development. It plays a significant role in advancing wearable FET biosensor technologies and translating them into commercial markets. Regulatory approval is a crucial hurdle that must be overcome to ensure compliance with relevant regulations and standards. Clinical validation is also critical to demonstrate the accuracy and effectiveness of wearable FET biosensors in clinical settings, and to gain acceptance from healthcare professionals and regulatory bodies. User acceptance is another important consideration, as wearable FET biosensors should be designed with user-friendly interfaces and comfortable form factors to promote engagement and adherence to monitoring protocols. Effective data management strategies are necessary to handle the substantial volume of data generated by wearable FET biosensors, while at the same time ensuring data privacy, integrity, and accessibility. Moreover, the wearable biosensor market is highly competitive. Companies need to differentiate themselves through unique features, superior performance, and competitive pricing to gain a foothold and thrive in the market. In conclusion, wearable biosensors hold great potential to revolutionize healthcare and wellness [24]. However, their successful integration and widespread adoption necessitate multidisciplinary collaboration and innovation to overcome technical, clinical, and commercial barriers. By addressing these challenges and leveraging emerging opportunities, wearable FET biosensors can drive transformative advancements in healthcare, contributing to improved patient outcomes and overall well-being.

## Perspective and conclusion

Wearable field-effect transistor (FET) biosensors represent a promising avenue for the future of healthcare monitoring. Our comprehensive review examined the recent progress made in FET sensor technology and explored its potential applications in diagnostics. By enabling non-invasive monitoring biomarkers in sweat, tears, saliva, and interstitial fluid, these devices provide real-time health insights that are easy to access, reliable, and cost-effective. Over time, these devices have been developed with various technologies including gating, material enhancements, semiconductor layers, and functionalization methods. These innovations have improved sensor sensitivity, reduced detection limits, and expanded the range of conditions they can address. We also present fabrication techniques as well as sensing probes including enzyme, antibody/nanobody, aptamer and ion-selective membrane. The ongoing development of continuous and non-invasive health monitoring holds the promise of increased accessibility and usability for individuals, with heightened accuracy, available anytime and anywhere.

Despite their numerous advantages and the corresponding potential, current research studies largely remain confined to laboratory settings. However, there is an expectation that these developments will eventually transition into practical applications. We anticipate that with further development and refinement, these biosensors will become integral parts of our daily lives. As these devices become more user-friendly and accessible, individuals will have the power to monitor their health continuously, enabling early detection of health issues and timely interventions. In the near future, the advances in novel probe technologies such as aptamers and nanobodies holds the promise of detection of diverse diseases with improved sensitivity and detection limits. Moreover, the integration of multifunctional FET biosensors with predictive machine learning technologies such as time-series analytics [185–187], has the potential to elevate sensing capabilities. This integration will not only improve the accuracy and timeliness of disease assessments but also enable personalized health predictions. Additionally, the seamless integration of electronics components such as amplifier and analog/digital coverter links sensors with wireless devices such as wristwatches, smartphones, iPads and laptops. This integration will result in a more user-friendly and intuitive interface, allowing users to effortlessly access and interpret their health data. In this regard, addressing ethical and privacy concerns will be paramount. Striking the right balance between data collection and individual privacy is a challenge that needs ongoing attention. Researchers, policymakers, and technology companies must collaborate to establish clear guidelines and standards for data security and privacy in the context of wearable health monitoring.

In conclusion, wearable FET biosensors have the potential to revolutionize healthcare and wellness by providing real-time, non-invasive monitoring of biomarkers. Addressing the challenges discussed here through multidisciplinary collaboration will be key to achieve this goal.

## Fundings

T.T.H. Nguyen acknowledges funding from Australian Academy of Technological Sciences and Engineering. N.T. Nguyen and M.A. Huynh acknowledge funding from Australian Research Council (ARC) Discovery Project (DP220100261) and Laureate Fellowship (FL230100023). T.K. Nguyen acknowledges funding from Griffith University Postdoctoral Fellowship and DE240100408. M.C.N acknowledges funding from Griffith University Higher Degree Research Scholarship.

## Data Availability

Not applicable.
